# Comparison of nalbuphine and sufentanil for colonoscopy: A randomized controlled trial

**DOI:** 10.1371/journal.pone.0188901

**Published:** 2017-12-12

**Authors:** Chaoyi Deng, Xiao Wang, Qianmei Zhu, Yanming Kang, Jinlin Yang, Heng Wang

**Affiliations:** 1 Department of Anesthesiology, Sichuan University West China Hospital, Chengdu, Sichuan, China; 2 Department of Gastroenterology, Sichuan University West China Hospital, Chengdu, Sichuan, China; University Hospital Llandough, UNITED KINGDOM

## Abstract

**Objectives:**

Nalbuphine is as effective as morphine as a perioperative analgesic but has not been compared directly with sufentanil in clinical trials. The aims of this study were to compare the efficacy and safety of nalbuphine with that of sufentanil in patients undergoing colonoscopy and to determine the optimal doses of nalbuphine in this indication.

**Methods:**

Two hundred and forty consecutive eligible patients aged 18–65 years with an American Society of Anesthesiologists classification of I–II and scheduled for colonoscopy were randomly allocated to receive sufentanil 0.1 μg/kg (group S), nalbuphine 0.1 mg/kg (group N1), nalbuphine 0.15 mg/kg (group N2), or nalbuphine 0.2 mg/kg (group N3). Baseline vital signs were recorded before the procedure. The four groups were monitored for propofol sedation using the bispectral index, and pain relief was assessed using the Visual Analog Scale and the modified Behavioral Pain Scale for non-intubated patients. The incidences of respiratory depression during endoscopy, nausea, vomiting, drowsiness, and abdominal distention were recorded in the post anesthesia care unit and in the first and second 24-hour periods after colonoscopy.

**Results:**

There was no significant difference in analgesia between the sufentanil group and the nalbuphine groups (p>0.05). Respiratory depression was significantly more common in group S than in groups N1 and N2 (p<0.05). The incidence of nausea was significantly higher in the nalbuphine groups than in the sufentanil group in the first 24 hours after colonoscopy (p<0.05).

**Conclusions:**

Nalbuphine can be considered as a reasonable alternative to sufentanil in patients undergoing colonoscopy. Doses in the range of 0.1–0.2 mg/kg are recommended. The decreased risks of respiratory depression and apnea make nalbuphine suitable for patients with respiratory problems.

## Introduction

Colonoscopy is now considered the “gold standard” for diagnosing pathologies of the colon and rectum, and is the primary modality used to screen for colorectal cancer [1]. However, colonoscopy involves air insufflation and insertion of instruments, is generally perceived by patients as painful, and is poorly tolerated without sedation [2]. Therefore, sedation and analgesia are widely accepted by patients and considered by many gastroenterologists as an integral component of the endoscopic examination. An effective analgesic agent for perioperative pain that does not produce significant respiratory depression (RD) would be useful for perioperative pain control during a colonoscopy procedure.

Nalbuphine hydrochloride is a mixed agonist-antagonist opioid with a duration of action of approximately 3–6 hours. It is chemically related to both the agonist analgesic oxymorphone and the antagonist naloxone, and acts as an antagonist at the μ receptor and as an agonist at the κ receptor, resulting in analgesia and sedation with minimal effects in the cardiovascular system [3]. Any slight RD that occurs would be restricted by a ceiling effect [4]. Other proposed advantages of nalbuphine as an agonist-antagonist opioid include a lower incidence of side effects (e.g., nausea and vomiting) than that with other opioid analgesics [5]. Further, nalbuphine is superior in treating opioid-induced pruritus without affecting analgesia [6].

Sufentanil, in contrast, is a highly lipophilic opioid fentanyl analog that is commonly used for surgical analgesia. However, sufentanil is associated with an increased risk of hypoxemia and apnea [3], which is particularly undesirable for patients and anesthesiologists in the outpatient setting. Nalbuphine is as effective as morphine for perioperative analgesia [7] but has not been compared directly with sufentanil in clinical trials.

The aims of this study were to compare the analgesic efficacy and safety of nalbuphine with that of sufentanil in patients undergoing colonoscopy and to determine the optimal doses of nalbuphine for colonoscopy.

## Materials and methods

This prospective, randomized, double-blind, clinical trial was approved by the ethics committee at West China Hospital, Sichuan University, in February 2016 and was registered with the Chinese Clinical Trial Registry (ChiCTR-IPR-16009184; http://www.chictr.org.cn) before its initiation in September 2016. The trial included 240 inpatients and outpatients who underwent colonoscopy at our institution from September 2016 to November 2016. Details of the study protocol can be found on http://dx.doi.org/10.17504/protocols.io.iq4cdyw.[PROTOCOL DOI]

The inclusion criteria were as follows: age 18–65 years; body mass index 18.5–30 kg/m^2^; American Society of Anesthesiologists classification I–II; and duration of colonoscopy <30 minutes for ensuring a standardized duration, which avoids excessive dosage and high risk for side effects.

Patients were excluded if they had a history of abnormal recovery from anesthesia, a heart rate on electrocardiography of <60 beats/min; systolic blood pressure (SBP) >180 mmHg or <90 mmHg, acute airway inflammation in the previous 2 weeks, neuromuscular disease, a possible or confirmed difficult airway, a suspected history of abuse of narcotic analgesics or sedatives, a history of allergy to propofol or opioids, or inability to communicate.

After obtaining written informed consent, the patients were randomly assigned to one of four groups (by opening of a sealed allocation envelope that contained the group randomization number produced by the blockrand package R3.1.1 [R Foundation for Statistical Computing, Vienna, Austria] in which the block size is 8) without stratification and received either sufentanil 0.1 μg/kg (group S), nalbuphine 0.1 mg/kg (group N1), nalbuphine 0.15 mg/kg (group N2), or nalbuphine 0.2 mg/kg (group N3). To blind the anesthesiologist to study group allocation, the doses used in the four groups were prepared in the same empty 10-mL syringes as 1 μg/mL, 1 mg/mL, 1.5 mg/mL, and 2 mg/mL by the nurse. In this way, all patients, the anesthesiologist, and the gastroenterologists were blinded to group information.

Patients in all groups received propofol for sedation and were then monitored for sedation depth using the bispectral index (BIS). Propofol was initially administered at a rate of 1 mL (10 mg)/5 seconds to a maximum dose of 4 mL (40 mg) if body weight was <60 kg or 5 mL (50 mg) if body weight was >60 kg. After each bolus infusion, a waiting period, typically 30–60 seconds, was used to observe and assess whether the drug had completely taken effect, judging by the BIS value falling below 80 and absence of the eyelash reflex. Additional doses (20–30 mg) of propofol were administered if the patient started to move or if the BIS value started rising to 80. The targeted sedation depth was moderate-to-deep, i.e., a stable BIS score between 60 and 80 during the procedure.

Baseline vital signs were recorded immediately before the procedure. All patients received supplemental oxygen intranasally (5 L/min) and underwent continuous monitoring of heart rate (three-lead electrocardiography), oxygen saturation (pulse oximetry), blood pressure (automated blood pressure cuff, serial measurements every 3 minutes), and BIS (BIS VISTA™ monitoring system, Aspect Medical Systems Inc., Norwood, MA, USA) at 1-minute intervals for the first 3 minutes after induction and every 3 minutes thereafter. The respiratory rate and end-tidal CO_2_ were recorded using a nasal cannula (Microstream® end-tidal CO_2_ circuit, Medtronic, Dublin, Republic of Ireland).

Pain intensity was evaluated using the modified Behavioral Pain Scale (BPS) for non-intubated patients (BPS-NI) [8] during endoscopy as the primary outcome variable. The modified BPS-NI is based on a summed score of three items, i.e., facial expression, movements of the upper and lower limbs, and vocalizations. A total score on the modified BPS-NI of >5 meant that the patient experienced intolerable pain during the colonoscopy procedure (Table 1). Pain relief was also measured using the Visual Analog Scale (VAS), which consists of a 10-cm horizontal line, the left end representing “no pain” (0 cm) and the right end representing “the worst imaginable pain” (10 cm). The patients were instructed to draw a vertical mark on the line to indicate the previous intensity of pain as the baseline before the procedure. After the procedure, we reassessed the pain level; the assessments were performed when the patients awoke in the post anesthesia care unit (PACU) who were not informed of the previous VAS score.

**Table 1:** Modified Behavioral Pain Scale score for non-intubated patients [8].

Item	Description	Scroe
Facial expression	Relaxed	1
	Partially tightened (brow lowering)	2
	Fully tightened (eyelid closing)	3
	Grimacing (folded cheek)	4
Upper or lower limbs	No movement	1
	Partially bent upper or lower limbs	2
	Fully bent with finger flexion	3
	Permanently retracted, affecting inspection	4
Vocalization	No pain vocalization	1
	Moaning not frequent (≤3/min) or prolonged (≤3 s)	2
	Moaning frequent (>3/min) and prolonged (≥3s)	3
	Howling or verbal complaint including “ow, ouch”	4

The total propofol dose was also documented. After the procedure, the patients were taken to a recovery room where blood pressure, blood oxygen saturation level (SpO_2_), and electrocardiographic parameters were monitored until discharge. After discharge, each patient received a follow-up telephone call during the first and second 24-hour periods after colonoscopy. Common side effects, including drowsiness, nausea, vomiting, and abdominal distension, were recorded. Data for patients who were unable to be contacted by follow-up telephone call were still included in the final analysis as part of the full analysis set, with the exception of side effects reported in the first and second 24-hour periods after colonoscopy. Thus, if patients were missing data on adverse events in the first and second post-discharge 24-hour periods, they were considered to be lost to follow-up. All patients (except those excluded on the basis duration of colonscopy) had data for the main outcomes, modified BPS-NI and VAS, before and after the procedure. All the data were recorded by the anesthesiologist.

Respiratory depression was considered to be significant when SpO_2_ was 90%, end-tidal CO_2_ was >50 mmHg at any time, respiratory rate was <6 breaths/minute, or when airway obstruction with cessation of gas exchange was observed at any time (noted by an absent end-tidal CO_2_ waveform) [9]. Airway maneuvers (i.e., jaw thrust and chin lift) would be manipulated in the event of RD. A decrease in SBP to <90 mmHg was considered to indicate hypotension. Ephedrine 3–5 mg was administered to treat arterial hypotension, which was defined as SBP of 80 mmHg or a reduction in SBP of >30% compared with baseline. Bradycardia was defined as a reduction in heart rate to 60 beats/minute; intravenous atropine 0.3–0.5 mg was administered in cases where the heart rate decreased to <50 beats/minute.

### Statistical analysis

The primary outcome variable was the modified BPS-NI score. Before the trial, we conducted a preliminary study using the same protocol that included 77 patients (20 in group S, 18 in group N1, 19 in group N2, and 20 in group N3). According to the preliminary test results, the proportion of patients with a modified BPS-NI score <5 in group S was 94.1% and the proportion of patients with a modified BPS-NI score <5 in groups N1, N2, and N3 was 88.24%, 68.75%, and 75%, respectively. Assuming a 1-β value of 0.9 and an α test level of 0.05, we needed a sample size of 207 according to the PASS 11 (NCSS, LLC., Kaysville, UT, USA) method for multiple sets of sample rates with an effect size (W) of 0.2620 (W = SQRT[(ChiSquare)/N], N = 77) [10], based on the difference of four groups. Allowing for a dropout rate of 10%, we calculated that 240 cases would need to be enrolled. The statistical calculations were performed using R3.2.1. The distribution of the data was checked for normality using the Shapiro-Wilk test. Depending on the data distribution, analysis of variance or the Kruskal-Wallis test was used for all independent continuous variables. Multiple comparisons were made using the Tukey’s honest significant difference test or the Nemenyi test, and the data were presented as the 95% family-wise confidence level. The data are presented as the mean and standard deviation when the distribution was normal. Categorical variables are presented as proportions (%) and were compared using Fisher’s exact test or the chi-squared test. Trends in the nalbuphine doses were evaluated using the chi-square test for trend. A p-value of <0.05 was considered to be statistically significant.

## Results

**Fig 1:**
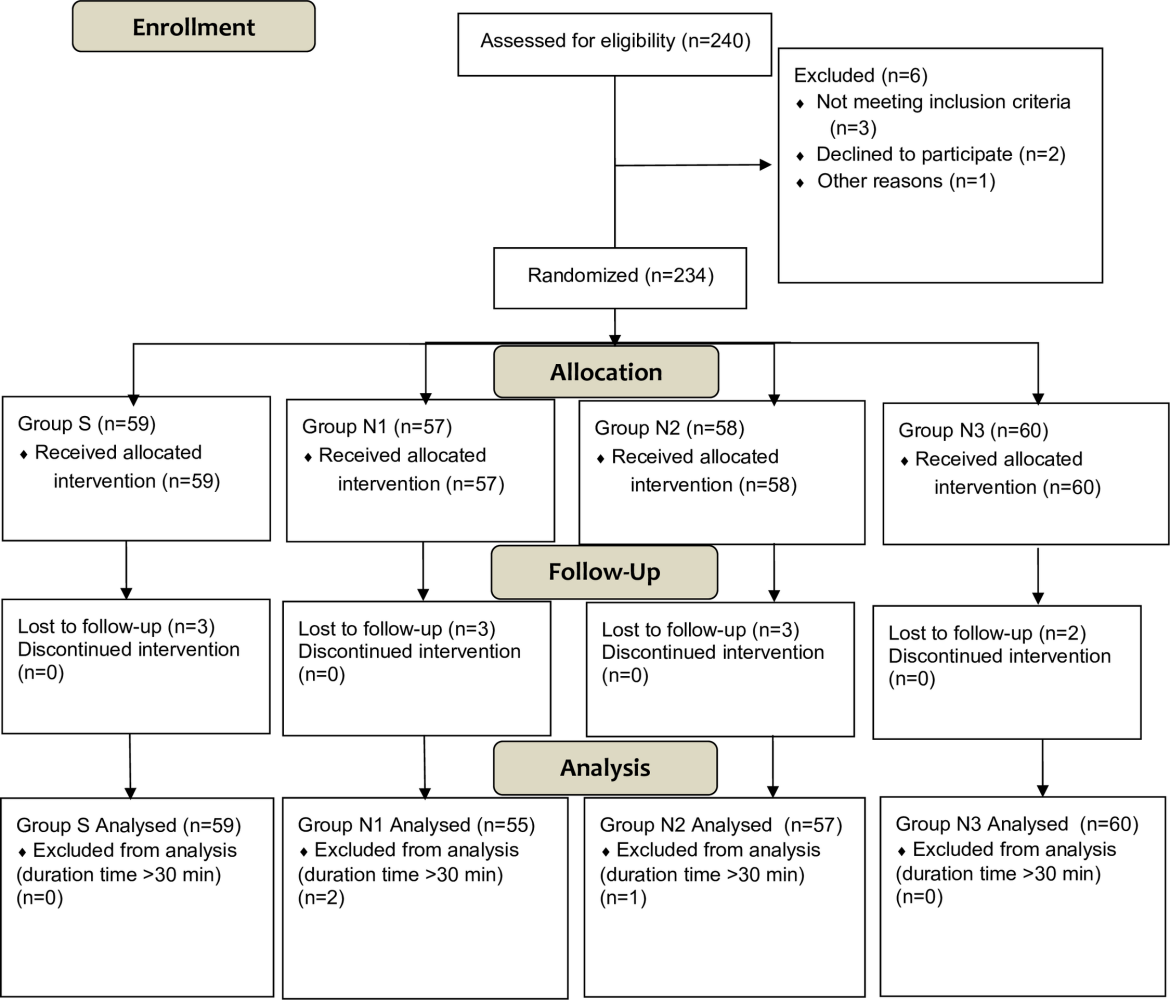
Flow chart showing the study design.

Two hundred and forty patients were assessed for eligibility. Six were excluded, leaving 234 eligible patients who were randomly allocated to group S (n = 59), group N1 (n = 57), group N2 (n = 58), or group N3 (n = 60). Eleven patients were unable to be contacted by follow-up telephone call so were considered lost to follow-up, and in three patients, the duration of colonoscopy was more than 30 minutes (Fig 1).

The patient demographic data for the study groups are presented in Table 2. No statistically significant demographic differences were noted between the four groups.

**Table 2:** Patient demographic characteristics.

	Group S	Group N1	Group N2	Group N3
Patients, n	59	55	57	60
Male/female, n	23/36	28/27	30/27	35/25
Mean age, years (± SD)	44.19 ± 11.67	44.71 ± 12.63	44.93 ± 10.15	44.98 ± 10.79
Mean BMI (± SD)	22.41 ± 2.94	22.72 ± 2.91	23.07 ± 2.25	23.48 ± 2.75
ASA classification				
I	29	23	23	26
II	30	32	34	34

ASA, American Society of Anesthesiologists; BMI, body mass index; SD, standard deviation

The intensity of pain during colonoscopy, as evaluated by the modified BPS-NI, was not significantly different between the groups (Table 3). The analgesic effects of nalbuphine at these doses did not differ significantly from those of sufentanil (p>0.05). A test for trend was also performed but did not show a dose-response relationship in terms of pain in the nalbuphine groups (p>0.05). There were no significant differences between the pre-intervention and post-intervention VAS scores within groups or the pre-examination and post-examination VAS scores among the groups. Further, there were no significant differences between the pre- to post-examination changes in scores among the four groups (p>0.05; Table 4).

**Table 3:** Occasions when the modified BPS-NI score was more than 5 during colonoscopy.

Occasions when BPS-NI score was >5	Group S	Group N1	Group N2	Group N3	p-value
0 (%)	56 (94.9)	45 (82.8)	51 (89.5)	56(93.3)	0.11
1 (%)	2 (3.4)	10 (18.2)	5 (8.8)	2(3.3)	
2 (%)	1 (1.7)	0 (0)	1 (1.7)	1(1.7)	
3 (%)	0 (0)	0 (0)	0 (0)	1(1.7)	

BPS-NI, Behavioral Pain Scale for non-intubated patients

**Table 4:** Visual analog scale scores.

	Group S	Group N1	Group N2	Group N3	p-value
Pre-examination, mean (± SD)	0.02 ± 0.13	0.16 ± 0.50	0.11 ± 0.36	0.10 ± 0.40	0.24
Post-examination, mean (± SD)	0.02 ± 0.13	0.09 ± 0.48	0.12 ± 0.38	0.10 ± 0.35	0.18
p-value*	1	0.28	0.77	1	

*There were no significant differences between the pre- to post-examination changes in scores among the four groups. SD, standard deviation

**Fig 2:**
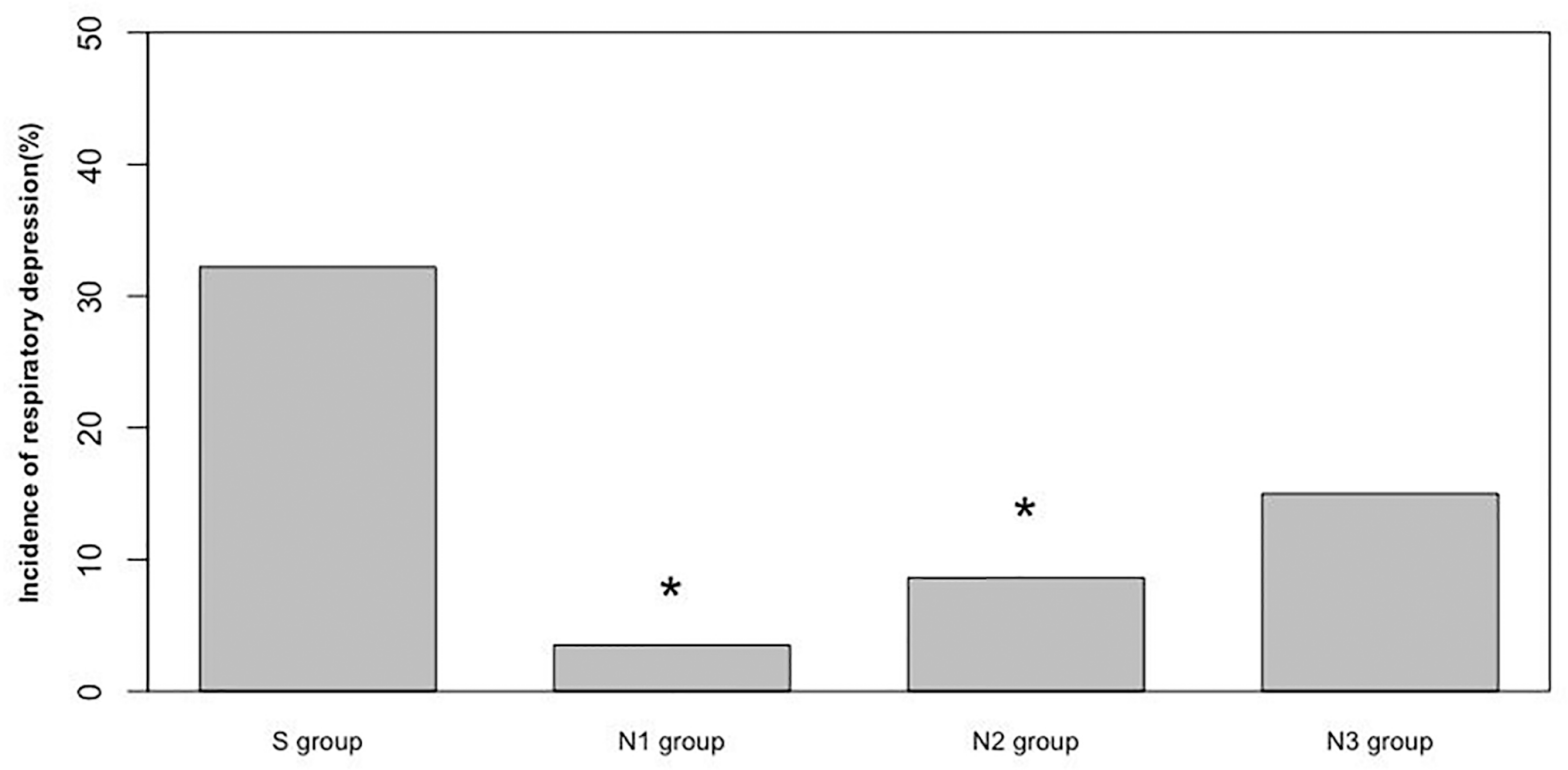
Incidence of respiratory depression. *Significantly lower RD than group S.

Using our measurement criteria, RD was detected as noted in Table 5 and Fig 2. The incidences of absent end-tidal CO_2_, SpO_2_ <90%, and RR <6 breaths/min were significantly lower in the nalbuphine group than in the sufentanil group. Only patients in groups N1 and N2 showed significantly lower RD than group S (p<0.05). When a test for trend was performed, there was a dose-response relationship in terms of RD in the nalbuphine groups (p<0.05). The incidence of RD was increased when the dose of nalbuphine was increased.

**Table 5:** Patients meeting the criteria for respiratory depression.

Criteria for RD	Group S	Group N1	Group N2	Group N3	p-value
End-tidal CO_2_ >50 mmHg (%)	2 (3.4)	0 (0)	2 (3.5)	2 (3.3)	0.41
Absent end-tidal CO_2_ waveform (%)	11 (18.6)	2 (3.6)*	2 (3.5)*	6 (10.0)	0.02^1^
SpO_2_ <90% (%)	9 (15.3)	0 (0)*	2 (3.5)	4 (6.7)	0.009^1^
RR <6 breaths/minute (%)	11 (18.6)	2 (3.5)*	1 (1.8)*	3 (5.0)	0.002^1^

*Significantly lower RD than group S.

^1^p<0.05 with Bonferroni correction.

SpO_2_, blood oxygen saturation level; RR, respiratory rate

The mean (± standard deviation) total propofol dose administered was 130.56 ± 41.05 mg in group S, 146.09 ± 37.56 mg in group N1, 126.28 ± 37.28 mg in group N2, and 123.35 ± 38.15 mg in group N3 (N1 vs. N2, p<0.05 and N1 vs. N3, p<0.01, Tukey’s honest significant difference test). There were no statistically significant differences between the other two groups. The total dose of propofol decreased when the nalbuphine dose was increased.

**Fig 3:**
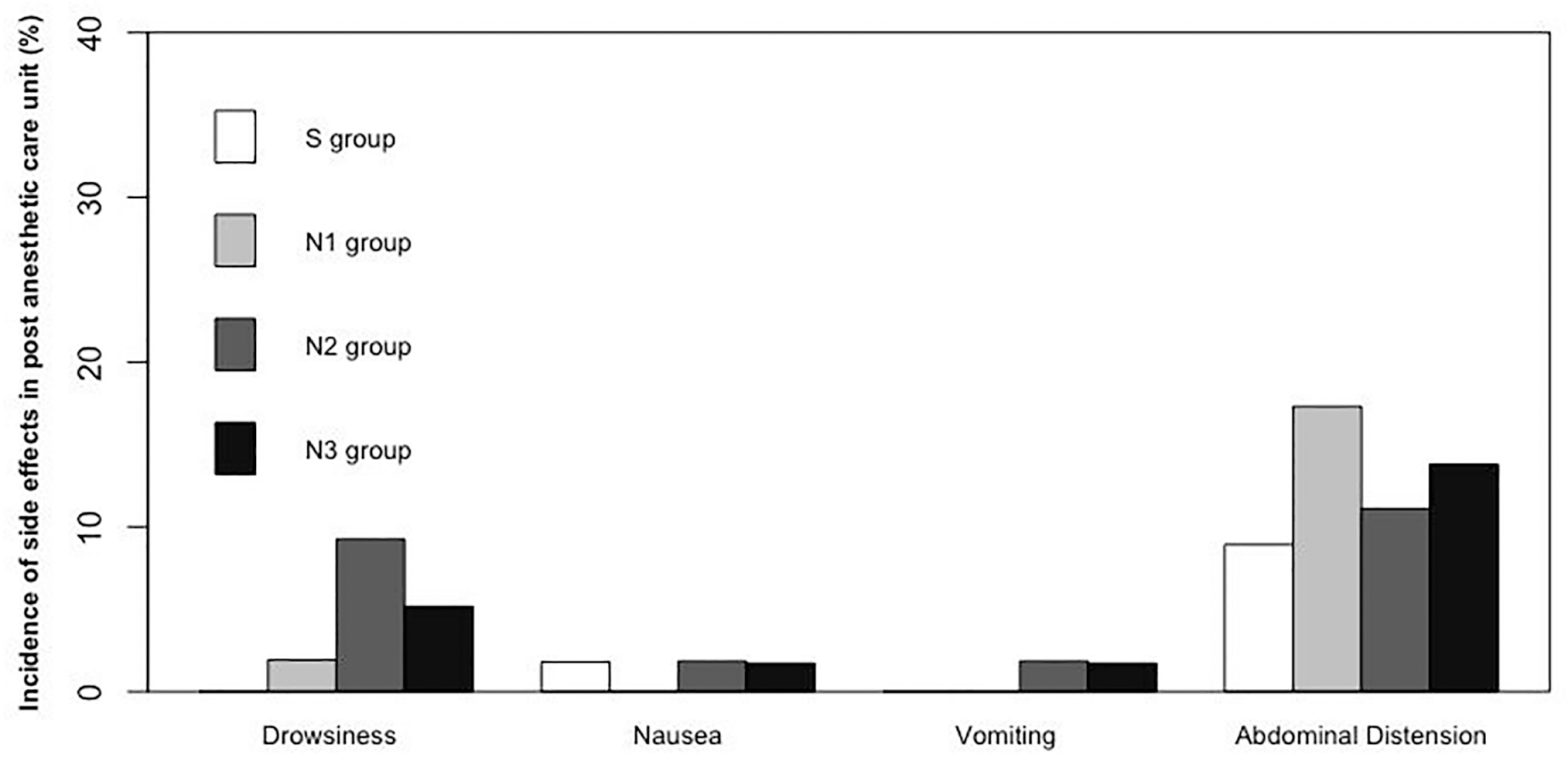
Side effects observed in the post anesthesia care unit.

Fig 3 shows the most common side effects, including drowsiness, nausea, vomiting, and abdominal distension, encountered in the PACU. There were no statistically significant differences between the groups (p>0.05). Table 6 shows these common side effects in the first 24-hour and second 24-hour periods after colonoscopy.

**Table 6:** Side effects in the first and second 24-hour periods after colonoscopy.

		Group S	Group N1	Group N2	Group N3	p-value
	Drowsiness	0	0	4 (7.3)	3 (5.2)	0.05
**Side effects**	Nausea	2 (3.6)	12 (23.1)*	14 (25.9)*	18 (31.0)*	0.002^1^
**in the first 24-hour period**	Vomiting	2 (3.6)	7 (13.5)	7 (15.5)	12 (20.7)	0.06
**after colonoscopy (%)**	Abdominal distension	4 (7.1)	3 (5.8)	5 (9.3)	3 (5.2)	0.84
	Drowsiness	0	0	0	0	1
**Side effects**	Nausea	0	0	0	2 (3.5)	0.25
**in the second 24-hour period**	Vomiting	0	0	0	1 (1.7)	1
**after colonoscopy** **(%)**	Abdominal distension	0	0	1 (1.9)	0	0.48

*Patients in the nalbuphine groups had a significantly higher incidence of nausea in the first 24 hours after colonoscopy than those in the sufentanil group.

^1^p<0.05 with Bonferroni correction.

## Discussion

The results of this study demonstrated that the three dose strengths of nalbuphine produced analgesia that was not significantly worse than that achieved by sufentanil. The BPS has been shown to dependably identify the extent of analgesia produced by fentanyl and other analgesics [11]. It is now well established that accurate assessment is the basis for effective pain management. Patients’ manifestations of pain include vocalizations, movement and mobility, facial expressions, and mood and behaviors, which are also used as behavioral indicators to assess pain in patients who are non-verbal [12]. Behavioral pain assessment tools recommended by the American Society for Pain Management Nursing may help us to recognize patients who are in pain but are unable to self-report. The BPS-NI is an adaptation of the original BPS that is used for non-intubated critically ill patients and could also be used for clinical research and during nociceptive procedures [13]. Comparison of the analgesic efficacy of nalbuphine and sufentanil for painful incidents during colonoscopy showed that both drugs provided adequate analgesia; most patients showed only slight or no response to the pain. It is noteworthy that all three doses of nalbuphine used in the study achieved pain relief similar to that achieved by sufentanil. Previous studies by Waye and Braufeld concluded that nalbuphine does not appear to be as effective as meperidine for relieving the discomfort of colonoscopy [14]. However, given the small sample size in their study, it is hard to reach any definitive conclusions. Other researchers demonstrated that visceral analgesia mediated by κ opioid receptor agonists is particularly effective [15]. In our study, nalbuphine proved to be an acceptable alternative to sufentanil and provided good analgesia of adequate duration.

Perioperative RD has always been a major factor limiting the use and safety of intraoperative opioids. Many studies have suggested that end-tidal CO_2_ monitored by nasal cannula is an excellent way of assessing RD during procedural sedation and analgesia [16]. Supplemental oxygen improved oxygenation in patients with decreased oxygen saturation levels in whom subclinical RD might not be able to be detected, but the increased end-tidal CO_2_ persisted despite the correction of oxygen saturation [17]. Thus, in our study, we used a series of indicators to monitor RD. We observed that nalbuphine dosages of 0.1 mg/kg and 0.15 mg/kg were associated with significantly less RD when compared with sufentanil. This may due to the μ-selective opioid sufentanil showing high affinity for its binding sites, which would moderate their main action in the brainstem. Therefore, marked RD would be induced because of their close vicinity to the respiratory regulating centers in the brainstem. This is the reason why opioid-induced RD has always been considered to be related to agonism at the μ receptor. In contrast, because κ receptors are distributed mainly within the cortex [18], a weaker RD effect would be induced by nalbuphine. Previous research by Gal et al indicated that nalbuphine produced a ceiling effect for RD at doses above 0.15 mg/kg [19]. Another study showed that the dose-effect curve for RD was flatter for nalbuphine than for morphine, and the maximum RD effects of nalbuphine occurred after a dose of 30 mg/70 kg [20]. It is possible that the mild RD effect of nalbuphine reflects the activity of this drug as a pure antagonist at the μ receptor and as an agonist at the κ receptor [21].

Another outcome observed in the present study was the lower mean dose of propofol in group N3 than in N2. This suggests that nalbuphine induces a sedative effect because of its action on the κ receptor, which could result in a reduction in the propofol dose required. Previous work has also shown that the total propofol dose were significantly lower in a group that received nalbuphine and propofol when compared with a group that received propofol alone [22].

We also found that the incidence of nausea in the 24 hours after colonoscopy was significantly higher in all the nalbuphine groups than in the sufentanil group. There were several factors that influenced the incidence of postoperative emetic episodes [23]. Several risk factors have been validated, including female sex, age <50 years, a history of postoperative nausea and vomiting, an opioid used in the PACU, and nausea in the PACU [24]. Garfield et al demonstrated that patients receiving nalbuphine 300 μg/kg or 500 μg/kg had a significantly higher incidence of nausea than patients receiving fentanyl and there was a suggestion of a dose-effect relationship [25]. However, a meta-analysis of randomized controlled trials showed that the incidences of nausea and vomiting were significantly lower in patients who received nalbuphine than in those who received morphine [5]. In our study, almost all the patients who developed nausea responded well to anti-emetic agents. Thus, preventive anti-emetic agents or low-dose nalbuphine could be given, especially in the subset of high-risk patients.

In conclusion, the results of this study confirm that nalbuphine is a reasonable alternative to sufentanil for intravenous analgesia in patients undergoing colonoscopy. Nalbuphine also produces less RD and has a decreased risk of apnea during colonoscopy procedures. However, in this study, incidence of nausea was significantly higher in the nalbuphine group in the first post-discharge 24-hour period.

## Supporting information

S1 File Supporting data. (XLSX)

S2 File Protocol in chinese. (DOC)

S3 File Protocol. (DOC)

S4 File Consort checklist. (DOC)

S5 File Ethics approval. (DOC)

S1 Fig Original ethics approval. (JPG)
