# Supplementary material for: Comparison of nalbuphine and sufentanil for colonoscopy: A randomized controlled trial
Source: PLoS One. 2017 Dec 12;12(12):e0188901. doi: 10.1371/journal.pone.0188901 (PMC5726642; doi:10.1371/journal.pone.0188901)
Supplement: S2 File — (DOC) [file pone.0188901.s002.doc]

ÁÙ´²ÑÐ¾¿·½°¸

ÄÔµçË«ÆµÆ×Ö¸Êý¼à²âÏÂÄÉ²¼·ÈÓëÊæ·ÒÌ«ÄáÓÃÓÚÎÞÍ´½á³¦¾µµÄµÈÐ§¼ÁÁ¿µÄÑÐ¾¿


°æ±¾£º       µÚ2.0°æ
ÈÕÆÚ£º       2015Äê11ÔÂ20ÈÕ


ÊÔÑéÖÐÐÄ£º      ËÄ´¨´óÑ§»ªÎ÷Ò½Ôº
ÑÐ¾¿¿ÆÊÒ£º      Âé×í¿Æ
ÑÐ¾¿¸ºÔðÈË£º      ÍõÏþ    ½ÌÊÚ


·½°¸ÕªÒª

·½°¸Ãû³Æ	ÄÔµçË«ÆµÆ×Ö¸Êý¼à²âÏÂÄÉ²¼·ÈÓëÊæ·ÒÌ«ÄáÓÃÓÚÎÞÍ´½á³¦¾µµÄµÈÐ§¼ÁÁ¿µÄÑÐ¾¿	
°æ±¾ºÅ/ÈÕÆÚ	µÚ2.0°æ  2015Äê11ÔÂ20ÈÕ	
ÊÔÑéÄ¿µÄ	±È½ÏÔÚÎÞÍ´½á³¦¾µÓ¦ÓÃS 0.1ug/kgÊæ·ÒÌ«Äá+±û²´·Ó£»N1 0.1mg/kgÄÉ²¼·È+±û²´·Ó£»N2 0.15mg/kgÄÉ²¼·È+±û²´·Ó£»N3 0.2mg/kgÄÉ²¼·È+±û²´·ÓËÄÖÖ¸øÒ©·½°¸ÓÐÐ§ÐÔµÄ±È½Ï	
ÊÔÑéÉè¼Æ	Ç°Õ°ÐÔ¡¢Ëæ»úÉè¼Æ
ÊÜÊÔÕß½«Ëæ»ú·ÖÅäÖÁËÄ×é	
²¡Àý×ÜÊý	240Àý	
ÑÐ¾¿ÆÚÏÞ	Õû¸öÑÐ¾¿Ô¤¼Æ3¸öÔÂ	
ÑÐ¾¿²¡Àý
Ñ¡Ôñ±ê×¼	ÈëÑ¡±ê×¼£º
1.	Äâ½øÐÐÎÞÍ´½á³¦µÄ»¼Õß£»
2.	18-65 ÖÜËê»¼Õß£¬ÐÔ±ð²»ÏÞ£»
3.	ÌåÖØÖ¸Êý£¨BMI£©18.5~30 kg/m2£»
4.	ÃÀ¹úÂé×íÒ½Ê¦Ð­»á£¨ASA£©·Ö¼¶ÎªⅠ~ II¼¶£»
5.	¼ì²éÊ±¼äÓ¦ÔÚ 30min ÒÔÄÚ£¨¿ªÊ¼Ê±¼äÍ³Ò»¶¨Îª·ÅÈë½á³¦¾µÖÁÈ¡³ö½á³¦¾µµÄÊ±¼ä£©£»
ÅÅ³ý±ê×¼£º
1.	ÓÐÑÏÖØµÄÐÄ¡¢ÄÔ¡¢·Î¡¢¸Î¡¢ÉöºÍ´úÐ»¼²²¡²¡Ê·Õß£»
2.	¼ÈÍùÓÐÒì³£ÊÖÊõÂé×í»Ö¸´Ê·Õß£»
3.	ÐÄµçÍ¼ÌáÊ¾£ºÐÄÂÊ<50 ´Î/min
4.	2 ÖÜÄÚÓÐºôÎüµÀ¼±ÐÔÑ×Ö¢ÇÒÎ´ÖÎÓú²¡Ê·£»
5.	ÊõÇ°¸ßÑªÑ¹»¼ÕßÊÕËõÑ¹>180mmHg»ò< 90mmHg£»
6.	ÓÐÉñ¾­¼¡ÈâÏµÍ³¼²²¡¡¢¾«Éñ¼²²¡Õß£» 
7.	»³ÒÉÓÐÀÄÓÃÂé×íÐÔÕòÍ´Ò©»òÕò¾²Ò©Õß£»
8.	Ô¤²â¿ÉÄÜ·¢Éú»òÔø·¢ÉúÀ§ÄÑÆøµÀÕß£»
9.	ÒÑÖª¶ÔÈé¼Á¡¢°¢Æ¬ÀàÒ©Îï¹ýÃôÕß£»
10.²»ÅäºÏÎÞ·¨¹µÍ¨Õß£»	
ÑÐ¾¿Ò©Îï	ÄÉ²¼·È×¢ÉäÒº£¨Èð¾²£©20mg/2ml
èÛéÚËáÊæ·ÒÌ«Äá×¢ÉäÒº   50ug/1ml
±û²´·Ó×¢ÉäÒº£¨µÃÆÕÀûÂé£©200mg/20ml	


ÊÔÑé·Ö×é	S×é 0.1ug/kgÊæ·ÒÌ«Äá+±û²´·Ó£»n=60
N1×é 0.1mg/kgÄÉ²¼·È+ ±û²´·Ó£» n=60
N2×é 0.15mg/kgÄÉ²¼·È+ ±û²´·Ó£» n=60
N3×é 0.2mg/kgÄÉ²¼·È+ ±û²´·Ó£»n=60	
ÑÐ¾¿²½Öè	ÔÚÊÜÊÔÕßÇ©ÊðÖªÇéÍ¬ÒâÊé²¢·ûºÏÈëÅÅ±ê×¼ºó£¬½«ÊÜÊÔÕßËæ»ú·ÖÅäÖÁËÄ×é£¬Ã¿×é¾ùÒ»ÏßÂé×íÒ½Éú°´·½°¸¸øÒ©¡£	
ÁÆÐ§¼°°²È«ÐÔÖ¸±ê	Ö÷ ÒªÆÀ ¼ÛÖ¸ ±ê 
ÓÐÐ§ÐÔÖ¸±ê£º»ùÓÚÈçÏÂ3Ïî±äÁ¿µÄ3·ÖÁ¿±í£¬ÆÀ¹À³¦¾µ²åÈë¡¢¹ýÆ¢Çú¡¢¹ý¸ÎÇúÌõ¼þ£¨Ë³Àû¶È£©
1. µÖ¿¹²åÈë£ºÎÞ/ÇáÎ¢/Ã÷ÏÔ£»2. Ãæ²¿±íÇé£ºÎÞ/ÇáÎ¢±ä»¯/Í´¿à£»3. Ö«Ìå/Í·²¿ÔË¶¯£ºÎÞ/ÇáÎ¢/Ã÷ÏÔ
´Î ÒªÆÀ ¼ÛÖ¸ ±ê 
1.	Ê±¼ä£º¿ªÊ¼¸øÊæ·ÒÌ«Äá»òÄÉ²¼·È¿ªÊ¼ÓÃÃë±í¼ÇÂ¼Ê±¼ä£¬¼ÇÂ¼ÓÕµ¼Ê±¼ä¡¢ËÕÐÑÊ±¼ä¡¢Õý³£Éú»îºÍ¹¤×÷
2.	±û²´·ÓÓÃÁ¿¡¢³¦Èä¶¯ÆµÂÊ
3.	ÉúÃüÌåÕ÷£ºÑªÑ¹£¨BP£©¡¢ÐÄÂÊ£¨HR£©¡¢ÂöÑõ±¥ºÍ¶È£¨SPO2%£©£¨ÓÕµ¼Ç°¡¢ÓÕµ¼ºó1min¡¢2min¡¢3min¡¢6min¡¢9min¡¢12min¡¢15min¡¢18min¡¢21min¡¢24min¡¢27min¡¢30min£©
4.	VASÆÀ·Ö£¨ÁôÖÃÕë´©´ÌÎª»ù´¡VAS£¬ÇåÐÑºóÔÙÆÀ¹À£©
5.	²»Á¼ÊÂ¼þ£º
      ÊõÖÐ£ºµÍÑªÑ¹¡¢µÍÑõ±¥ºÍ¶È¡¢ÆøµÀ¹£×è¡¢ºôÎüÒÖÖÆ¡¢ºôÎüÔÝÍ£¡¢ÐÄ¶¯¹ý»º¡¢ÐÄ¶¯¹ýËÙ¡¢Ìå¶¯
      Êõºó£ºÀä/ÈÈ¡¢¶ñÐÄ¡¢Å»ÍÂ¡¢Ø¬ÃÎ/ÐÀÔÃ¡¢ÊÈË¯/Ñ£ÔÎ¡¢Æ¤·ôðþÑ÷
6.	ÂúÒâ¶È£º»¼Õß£»ÄÚ¾µ²Ù×÷Õß£»Âé×íÒ½Éú	
Í³¼Æ·ÖÎö	1£®ÊÔÑéÊý¾ÝµÄÍ³¼Æ·ÖÎö
1.1 È« ·ÖÎö ¼¯£¨ FAS£¬ full analysis set£© £º°´ ITT£¨Intention-to-treat£©Ô­Ôò£¬ËùÓÐ¾­Ëæ»ú»¯·Ö×é£¬ÖÁÉÙÊ¹ÓÃ¹ýÒ»´ÎÑÐ¾¿Ò© Æ·¡¢ÇÒ¾ßÓÐÓÃÒ©ºóÆÀ¼ÛÊý¾ÝµÄ²¡Àý£¬¹¹³É±¾ÑÐ¾¿µÄ FAS¡£FAS ÖÐÁÆÐ§Ïà¹Ø²¿·ÖµÄÈ± Ê§Êý¾Ý½«²ÉÓÃÖ®Ç°×îºóÒ»´Î¹Û²âÊý¾Ý½á×ªµÄ·½·¨½øÐÐ²¹³ä¡£FAS ÊÇÁÆÐ§ÆÀ¼ÛµÄÖ÷ÒªÈËÈº
1.2 ·û ºÏ·½ °¸¼¯ £¨ PPS£¬ per protocol set £© £ºPPS ÊÜÊÔÕß·ûºÏÊÔÑé·½°¸¹æ¶¨µÄÈëÑ¡±ê×¼,Íê³ÉÈ«²¿¹Û²ìÆÚ¼Æ»®£»ÊÔÑéÆÚ¼äÎ´ Ê¹ÓÃ¿ÉÄÜÓ°ÏìÁÆÐ§ÆÀ¼ÛµÄÆäËûÒ©Îï»òÖÎÁÆ´ëÊ©¡£PPS ÊÇÁÆÐ§ÆÀ¼ÛµÄ´ÎÒªÈËÈº¡£
1.3°² È«ÐÔ ·ÖÎö Êý¾Ý¼¯ £¨ SS£¬ safety set £© £º¾­ÊÜÊÔÕßËæ»ú»¯·Ö×éºó£¬ÖÁÉÙÊ¹ÓÃÒ»´ÎÑÐ¾¿Ò©Îï¡¢¾ßÓÐÓÃÒ©ºó°²È«ÐÔÆÀ¼ÛÊý¾ÝµÄÈ«²¿²¡Àý£¬¹¹³É±¾ÑÐ¾¿µÄ°²È«ÐÔ·ÖÎöÈËÈº¡£
 2.Í³¼Æ·ÖÎö·½·¨
ÓÃ SPSS21.0 Í³¼ÆÈí¼þ½øÐÐÍ³¼Æ·ÖÎö¡£
»ùÏßÆÀ¼Û: ÓëÁÆÐ§ÆÀ¼ÛÏà¹ØµÄÖ¸±êÔÚÉ¸Ñ¡ÆÚËù»ñµÃµÄÊý¾Ý¶¨ÒåÎª»ùÏßÊý¾Ý¡£°´ FAS¡¢PPS ½øÐÐ»ùÏßÆÀ¼Û¡£·ÖÀà×ÊÁÏµÄ×é¼ä±È½Ï²ÉÓÃÖÈºÍ¼ìÑé£¨µÈ¼¶×ÊÁÏ£©¡£¼ÆÁ¿×ÊÁÏµÄ×é¼ä±È½Ï²ÉÓÃµ¥ÒòËØ·½²î·ÖÎö»òÖÈºÍ¼ìÑé¡£ P≤0.05 ÈÏÎªÓÅÐ§³ÉÁ¢¡£	

ÊÔÑéÁ÷³ÌÍ¼


           
Ä¿	Â¼    
1.ÊÔÑé±³¾°ÓëÄ¿µÄ	1
2.ÊÔÑé×ÜÌåÉè¼ÆÓë°²ÅÅ	2
2.1 ÊÔÑéÓÃÒ©	2
2.2 ÑÐ¾¿Ãû³Æ	2
2.3 ÑÐ¾¿»ú¹¹	2
2.4 ÑÐ¾¿Ê±¼ä	2
2.5 ÑÐ¾¿·½·¨	2
2.6 ÈëÑ¡¡¢ÅÅ³ýºÍÍË³ö±ê×¼
2.6.1 ÈëÑ¡±ê×¼	2
2.6.2 ÅÅ³ý±ê×¼	3
2.6.3 ÍË³ö±ê×¼	3
2.7 ÊÔÑéÈËÔ±ÒªÇó	4
2.8 ÊÔÑé·½·¨	4
2.8.1 ÊÔÑéÓÃÒ©	4
2.8.2 ÊÔÑé·Ö×é¼°¸øÒ©·½°¸	4
2.8.3  Î§ÊõÆÚ¹ÜÀí	5
2.9 ¹Û²ìÖ¸±ê	6
2.9.1 Ò»°ãÖ¸±ê	6
2.9.2 Ö÷ÒªÖ¸±ê	6
2.9.3 ´ÎÒªÖ¸±ê	6
2.9.4 ÊõÖÐ¹Û²ìÖ¸±ê	7
2.9.5 Êõºó¹Û²ìÖ¸±ê	8
3.ÁÙ´²ÊÔÑé¼ÇÂ¼	9
4.ÊÔÑéÊý¾ÝµÄÍ³¼Æ·ÖÎö	9
4.1 ·ÖÎöÈËÈº	9
	È«·ÖÎö¼¯£¨FAS£¬full analysis set£©	9
	·ûºÏ·½°¸¼¯£¨ PPS£¬per protocol set £©	9
4.1.3 °²È«ÐÔ·ÖÎöÊý¾Ý¼¯£¨ SS£¬safety set £©	9
4.2 ÁÆÐ§ºÍ°²È«ÐÔÖ¸±ê	9
4.2.1 Ö÷ÒªÆÀ¼ÛÖ¸±ê	9
4.2.2 ´ÎÒªÆÀ¼ÛÖ¸±ê	9
4.2.3 ÆäËûÆÀ¼ÛÖ¸±ê	9
4.3 Í³¼Æ·ÖÎö·½·¨	10
4.3.1 Ò»°ãÔ­Ôò	10
4.3.2 ²¡ÀýÌØÕ÷	10
4.3.3 ÁÆÐ§ÆÀ¼Û	10
4.3.4 °²È«ÐÔÆÀ¼Û	10
5.ÊÔÑé¹ÜÀí	10
6. ÁÙ´²ÊÔÑé¸ºÔðÈËÇ©×Ö±í	11
7.¸½Â¼	11
	
1.	ÊÔÑé±³¾°ÓëÄ¿µÄ 
½á³¦¾µÕïÁÆ¼¼ÊõÊÇÏÂÏû»¯µÀ¼²²¡×îÖ÷Òª¡¢×î¿É¿¿µÄ·½·¨£¬ÇÒ½á³¦¾µÏÂÈ¡»î¼ì¶ÔÔçÆÚÕï¶ÏºÍÖÎÁÆÏÂÏû»¯µÀÖ×ÁöÓÐÖØ´óÒâÒå¡£µ«½á³¦¾µ²Ù×÷Ê±¼ä³¤£¬´Ì¼¤½ÏÇ¿£¬ÓÈÆä³¦¹Ü×¢Æø¼°±»Ç£À­£¬Ò»·½ÃæÒýÆð¶ñÐÄ¡¢ÌÛÍ´£¬ÉõÖÁ³¦ñÈ»ò³¦¾·ÂÎ£¬Ê¹ÑªÑ¹Éý¸ß¡¢ÐÄÂÊÔö¿ì£¬ÉõÖÁÓÕ·¢ÐÄ½ÊÍ´¡¢ÐÄ¼¡¹£ËÀ¡¢ÄÔ×äÖÐ»òÐÄ²«ÖèÍ£µÈÑÏÖØ²¢·¢Ö¢£»ÁíÒ»·½Ãæ¸ø»¼Õß´øÀ´½ôÕÅ¡¢½¹ÂÇ¡¢¿Ö¾åÐÄÀí£¬ÉÙ²¿·Ö»¼Õß²»ÄÜÄÍÊÜºÍÅäºÏÍê³ÉÄÚ¾µ²Ù×÷£¬´Ó¶øÊ¹ÄÚ¾µÒ½Ê¦ÎÞ·¨Ã÷È·µØÕïÖÎÏà¹Ø¼²²¡¡£Ä¿Ç°ÎÒ¹úºÜ¶àµ¥Î»ÒÑ¾­¿ªÕ¹ÄÚ¾µÕïÁÆµÄÕò¾²/Âé×í£¬ÇÒÓÐÖð½¥ÍÆ¹ãµÄÇ÷ÊÆ¡£Ïû»¯ÄÚ¾µÕïÁÆµÄÕò¾²/Âé×íÊÇÖ¸Í¨¹ýÓ¦ÓÃÕò¾²Ò©ºÍÂé×íÐÔÕòÍ´Ò©µÈÒÔ¼°Ïà¹Ø¼¼Êõ£¬Ïû³ý»ò¼õÇáÄÚ¾µ¼ì²é»òÖÎÁÆ¹ý³ÌÖÐµÄÌÛÍ´¡¢¸¹ÕÍ¡¢¶ñÐÄÅ»ÍÂµÈÖ÷¹ÛÍ´¿àºÍ²»ÊÊ¸Ð£¬ÓÈÆä¿ÉÒÔÏû³ý»¼Õß¶ÔÔÙ´Î¼ì²éµÄ¿Ö¾å¸Ð£¬Ìá¸ß»¼Õß¶ÔÏû»¯ÄÚ¾µµÄ½ÓÊÜ¶È£¬Í¬Ê±ÎªÄÚ¾µÒ½Ê¦´´Ôì¸üºÃµÄÕïÁÆÌõ¼þ¡£[1]
Ä¿Ç°ÁÙ´²ÉÏ³£ÓÃÖÐ¶È»òÉî¶ÈÕò¾²£¬¼´¾²Âö×¢Éä·ÒÌ«Äá£¨30~50ug£©»òÊæ·ÒÌ«Äá£¨3~5ug£©ºÍ£¨»ò£©Ð¡¼ÁÁ¿µÄßä´ïßòÂØ£¨1~2mg£©£¬ÔÙ¸øÓè±û²´·Ó1~2mg/kg»òÒÀÍÐßäõ¥0.2~0.3mg/kg¡£»¼Õß×ÔÖ÷ºôÎüÂÔ»ºÂýµ«Æ½ÎÈ¡¢½ÞÃ«·´ÉäÏûÊ§¡¢È«Éí¼¡ÈâËÉ³Ú¡¢ÍÐÏÂò¢ÎÞ·´Ó¦Ê±¿ªÊ¼ÄÚ¾µ²Ù×÷¡£Èç¹ûÕïÁÆÊ±¼äÉÔ³¤»ò²Ù×÷´Ì¼¤½ÏÇ¿£¬¸ù¾Ý»¼ÕßÌåÕ÷ÈçºôÎü¼ÓÉî¡¢ÐÄÂÊÔö¿ì£¬ÉõÖÁÌå¶¯µÈ£¬¿ÉÃ¿´Î¾²Âö×·¼Ó±û²´·Ó0.2~0.5mg/kg»òÒÀÍÐßäõ¥0.1mg/kg£¬Ö±ÖÁ¼ì²é½áÊø¡£[1] ±û²´·Ó¶ÔÑ­»·¡¢ÉÏºôÎüµÀ·´ÉäÓÐÒÖÖÆ×÷ÓÃ¼°µ¼ÖÂ»¼ÕßÎÞÄ¿µÄÐÔÔË¶¯¡£ËùÒÔ£¬²»½¨Òé½«Ö®×÷ÎªµÄ¶ÀÁ¢ÓÃÒ©£¬ÁÙ´²ÉÏÁªºÏÓ¦ÓÃ°¢Æ¬ÀàÒ©ÎïÓë±û²´·ÓµÄ³É¹¦ÂÊ¸ß£¬µ«ºôÎüÒÖÖÆ¡¢ºôÎüÔÝÍ£¡¢µÍÑªÑ¹¡¢Í·ÔÎ¡¢¼ì²éºó¸¹ÕÍ¸¹Í´ÈÔÈ»ÊÇÖ÷ÒªµÄ¸±×÷ÓÃ¡£[2]
         ÑÎËáÄÉ²¼·È[(-)-17(»·¶¡»ù¼×»ù)-4,5α-»·ÑõÂð·Èà«-3,6α,14-Èý´¼ÑÎËáÑÎ]ÊÇκÊÜÌå¼¤¶¯¼Á/²¿·ÖμÊÜÌåÞ×¿¹¼ÁµÄÕòÍ´¼Á£¬ÌÛÍ´¿ØÖÆË®Æ½ÓëÂð·ÈÏàµ±£¬ÓÃÓÚÖÎÁÆºÍÔ¤·ÀÖÐ¶ÈÖÁÖØ¶ÈÌÛÍ´¡£ÄÉ²¼·ÈÎÞÐÄÑª¹Ü¸±·´Ó¦,ºôÎüÒÖÖÆÒàÇáÎ¢,²¢ÓÐ·â¶¥Ð§Ó¦¡£ÄÉ²¼·ÈÍ¨³£ 2¡« 3·ÖÖÓÆðÐ§,30·ÖÖÓ´ï·å×÷ÓÃ,¿ÉÎ¬³Ö 3¡« 6Ð¡Ê±ÕòÍ´£¬·â¶¥Ð§Ó¦µÄ¼ÁÁ¿Îª 0. 3¡« 0. 5mg /kg¡£
          ¹úÍâÑÐ¾¿±íÊ¾£¬ÄÉ²¼·È¸´ºÏÂð·ÈÇÊÄÚ×¢Éä¼õÉÙÁË°¢Æ¬ÀàÒ©ÎïÏà¹Ø¸±×÷ÓÃÇÒ²»Ó°ÏìÊõºóÕòÍ´¡£[3]Ò²¿ÉÒÔÁªÍ¬¸ß±ÈÖØ²¼±È¿¨ÒòÇÊÄÚ×¢ÉäÓÃÓÚÏÂ¸¹²¿¡¢ÃÚÄòÏµÍ³¼°ÏÂÖ«ÊÖÊõÖÐÄÉ²¼·ÈºÍ±û²´·ÓÓÕµ¼ÓÃÓÚºíÕÖÍ¨Æø²åÈë[2]¹úÄÚ¶ÔÄÉ²¼·ÈÑÐ¾¿ºÜÉÙ£¬1992ÄêÓáÑôÈÏÎªÄÉ²¼·È¿ÉÓÃÓÚÃÅÕïÊÖÊõÂé×í¡£[4]2011Äê²ÌÁ¢æºµÈ½øÐÐÁËÄÉ²¼·È×¢ÉäÒºÔÚ½¡¿µÊÜÊÔÕßÖÐµÄÒ©´ú¶¯Á¦Ñ§ÑÐ¾¿¡£[5]2013ÄêÀè»·µÈ¶Ô±ÈÁËµØ×ôÐÁ¡¢Âð·È¡¢ÄÉ²¼·È¶ÔÓÚÃÅÕï»¼ÕßÊõºóÕòÍ´Ð§¹û¡£[6]
ÄÉ²¼·È²»ÔÚÐË·ÜÂé×íÒ©Æ·ºÍ¾«ÉñÀàÒ©ÎïµÄÇåµ¥ÖÐ£¬¿É×÷Îª·ÇÐË·ÜÂé×íÒ©Æ·´¢´æ£¬²¢»ùÓÚ³£¹æ»ù´¡½øÐÐ´¦·½Ó¦ÓÃ¡£[7]ÕâÒ²ÓÐÍû½â¾öÁËÎÒ¹úÊÖÊõÊÒÍâÂé×íÒò·ÒÌ«Äá¼Ò×åµÄÏÞÖÆÊ¹ÓÃ¶øµ¥¶ÀÓÃ±û²´·ÓÕò¾²µÄÎÊÌâ¡£´ËÍâ£¬ÒòÆä¶ÀÌØµÄÕòÍ´ºÍÖÐ¶ÈÕò¾²×÷ÓÃ»úÖÆ£¬ÊõÇ°Ó¦ÓÃÄÉ²¼·È0.1-0.2mg kg-1¿É»º½âÌÛÍ´¡¢´øÀ´Õò¾²ºÍ°²¶¨µÄ×÷ÓÃ¡£
¹Ê¶ø±¾ÑÐ¾¿Ö÷Òª¹Û²ì¼°±È½ÏÔÚÎÞÍ´½á³¦¾µÓ¦ÓÃÊæ·ÒÌ«ÄáºÍÄÉ²¼·ÈµÄÕòÍ´Ð§¹û¼°°²È«ÐÔ£¬Ì½ÇóÄÉ²¼·ÈÓÃÓÚÎÞÍ´³¦¾µµÄ×îÊÊ¼ÁÁ¿¡£ÑÐ¾¿¹ý³ÌÖÐÊ¹ÓÃµÄ±û²´·ÓºÍÊæ·ÒÌ«Äá¾ùÊÇ¾­¹ý³¤ÆÚµÄÁÙ´²Ó¦ÓÃÖ¤Ã÷ÊÇ°²È«¿É¿¿µÄ¾­µäÂé×íÒ©Æ·£¬ÄÉ²¼·È×¢ÉäÒºÎª¹úÄÚÒÑÉÏÊÐÒ©Æ·£¬¾­ÁÙ´²Ó¦ÓÃÖ¤Ã÷ÊÇ°²È«¿É¿¿¡£
2.ÊÔÑé×ÜÌåÉè¼ÆÓë°²ÅÅ
2.1 ÊÔÑéÓÃÒ©
Êæ·ÒÌ«Äá×¢ÉäÒº£ºÓÉÒË²ýÈË¸£Ò©ÒµÓÐÏÞ¹«Ë¾Éú²ú£¬50ug/1ml¡£
ÄÉ²¼·È×¢ÉäÒº£ºÉÌÆ·Ãû³ÆÎªÈð¾²£¬ÓÉÒË²ýÈË¸£Ò©ÒµÓÐÏÞ¹«Ë¾Éú²ú£¬20mg/2ml¡£
±û²´·Ó×¢ÉäÒº£ºÉÌÆ·Ãû³ÆÎªµÃÆÕÀûÂé£¬ÓÉ°¢Ë¹Àû¿µÒ½Ò©ÓÐÏÞ¹«Ë¾Éú²ú£¬200mg/20ml
2.2 ÑÐ¾¿Ãû³Æ
ÄÔµçË«ÆµÆ×Ö¸Êý¼à²âÏÂÄÉ²¼·ÈÓëÊæ·ÒÌ«ÄáÓÃÓÚÎÞÍ´½á³¦¾µµÄµÈÐ§¼ÁÁ¿µÄÑÐ¾¿
2.3 ÑÐ¾¿»ú¹¹
ËÄ´¨´óÑ§»ªÎ÷Ò½Ôº
2.4 ÑÐ¾¿Ê±¼ä
¿ªÊ¼Ê±¼ä 2015 Äê 12  ÔÂ	½áÊøÊ±¼ä 2016 Äê	12  ÔÂ
2.5 ÑÐ¾¿·½·¨ 
Ç°Õ°ÐÔ¡¢Ëæ»ú¡¢Ë«Ã¤ÊÔÑé
2.6 ÈëÑ¡¡¢ÅÅ³ýºÍÌÞ³ý±ê×¼
2.6.1 Èë Ñ¡±ê ×¼ 
ÒÔÏÂÈÎºÎÒ»ÏîÎª“·ñ”£¬´ËÊÜÊÔÕß²»ÄÜ²Î¼ÓÊÔÑé£º
	Äâ½øÐÐÎÞÍ´½á³¦¾µµÄ»¼Õß£»
	18-65ÖÜËê»¼Õß£¬ÐÔ±ð²»ÏÞ£»
	ÌåÖØÖ¸Êý£¨BMI£©18.5~30 kg/m2£»
	ÃÀ¹úÂé×íÒ½Ê¦Ð­»á£¨ASA£©·Ö¼¶ÎªⅠ~ II¼¶£»
	¼ì²éÊ±¼äÓ¦ÔÚ 30min ÒÔÄÚ£¨Ê±¼äÍ³Ò»¶¨Îª³¦¾µ½øÈëÖÁÈ¡³öµÄÊ±¼ä£©£»
2.6.2 ÅÅ ³ý±ê ×¼ 
ÒÔÏÂÈÎºÎÒ»ÏîÎª“ÊÇ”£¬´ËÊÜÊÔÕß²»ÄÜ²Î¼Ó£º
1	ÓÐÑÏÖØµÄÐÄ¡¢ÄÔ¡¢·Î¡¢¸Î¡¢ÉöºÍ´úÐ»¼²²¡²¡Ê·Õß£»
2 ¼ÈÍùÓÐÒì³£ÊÖÊõÂé×í»Ö¸´Ê·Õß£»
3	ÐÄµçÍ¼ÌáÊ¾£ºÐÄÂÊ<50 ´Î/min£»
4	2 ÖÜÄÚÓÐºôÎüµÀ¼±ÐÔÑ×Ö¢ÇÒÎ´ÖÎÓú²¡Ê·£»
5	ÊõÇ°¸ßÑªÑ¹»¼Õß>180 »ò< 90mmHg£»
6	ÓÐÉñ¾­¼¡ÈâÏµÍ³¼²²¡¡¢¾«Éñ¼²²¡Õß£» 
7	»³ÒÉÓÐÀÄÓÃÂé×íÐÔÕòÍ´Ò©»òÕò¾²Ò©Õß£»
8	Ô¤²â¿ÉÄÜ·¢Éú»òÔø·¢ÉúÀ§ÄÑÆøµÀÕß£»
9	ÒÑÖª¶ÔÈé¼Á¡¢°¢Æ¬ÀàÒ©Îï¹ýÃôÕß£»
10²»ÅäºÏÎÞ·¨¹µÍ¨Õß£» 
2.6.3 ÍË³ö±ê×¼
2.6.3.1 ²¡ÀýÌÞ³ý±ê×¼
ÒÑÈë×é²¡Àýµ«·ûºÏÏÂÁÐÖ®Ò»Õß£¬Ó¦ÓèÌÞ³ý£º
1	²»·ûºÏÈë×é±ê×¼»ò·ûºÏÅÅ³ý±ê×¼µÄ»¼Õß£»ÎÞÈÎºÎ¼ì²â¼ÇÂ¼Õß£»
2	²¡Àý±¨¸æ±í¼ÇÂ¼²»ÍêÕû£¬ÒÔÖÂÎÞ·¨×÷ÓÐÐ§ÐÔÆÀ¼ÛÕß£»
	3  Ê§·Ã£»
4	½á³¦¾µÊ±¼ä´óÓÚ 30min µÄ»¼Õß¡£ 
ÌÞ³ýµÄ²¡ÀýÓ¦ËµÃ÷Ô­Òò£¬ÏàÓ¦Ò½ÁÆºÍÑÐ¾¿¼ÇÂ¼Ó¦±£Áô±¸²é¡£²»×÷ÁÆÐ§Í³¼Æ·ÖÎö£¬µ«ÖÁÉÙ½ÓÊÜÒ»´ÎÖÎÁÆ£¬ÇÒÓÐ°²È«¼ÇÂ¼Õß£¬ÊÓÇé¿ö¿É²Î¼Ó°²È«ÐÔ·ÖÎö¡£
2.6.3.2 ²¡ÀýÍÑÂä±ê×¼ 
ÒòÒÔÏÂÔ­ÒòÎ´Íê³ÉÁÙ´²ÊÔÑé·½°¸µÄÈë×é²¡ÀýÊÓÎªÍÑÂä£º
1 Òò²»Á¼ÊÂ¼þ²»ÄÜÍê³ÉÖÎÁÆÕß£»
2	ÑÐ¾¿ÕßÇëÆäÍË³ö£¨ÒÀ´ÓÐÔ²î¡¢ÑÏÖØ²»Á¼ÊÂ¼þ£©£»
3  ÑÐ¾¿ÆÚ¼äºÏ²¢Ê¹ÓÃ½ûÖ¹Ê¹ÓÃµÄÒ©Îï£¬ÒÔÖÂÎÞ·¨×÷³öÒ©Ð§ºÍ°²È«ÐÔÆÀ¼ÛÕß¡£
2.7 ÊÔÑéÈËÔ±ÒªÇó
	ÊÖÊõÒ½Ê¦¹Ì¶¨ 2 Ãû£»ÅäÒ©ÈËÔ±¹Ì¶¨1Ãû£»Âé×íÒ½Éú¹Ì¶¨1Ãû£»¹Û²ìÈËÔ±¹Ì¶¨ 1 Ãû
2.8 ÊÔÑé·½·¨
2.8.1 ÊÔÑéÓÃ Ò© 

Í¨ÓÃÃû	³§¼Ò	ÉÌÆ·Ãû	¹æ¸ñ	
èÛéÚËáÊæ·ÒÌ«Äá×¢ÉäÒº	ÒË²ýÈË¸£Ò©ÒµÓÐÏÞ¹«Ë¾		50ug/1ml	
ÄÉ²¼·È×¢ÉäÒº	ÒË²ýÈË¸£Ò©ÒµÓÐÏÞ¹«Ë¾	  Èð¾²	20mg/2ml	
±û²´·Ó×¢ÉäÒº	°¢Ë¹Àû¿µ¹«Ë¾	µÃÆÕÀûÂé	200mg/20ml	
2.8.2 ÊÔÑé·Ö×é¼°¸øÒ©·½°¸
»¼Õß°´ÕÕÈë×éµÄÏÈºóË³Ðò£¬ÔÙÒÀÕÕËæ»ú»¯·½·¨ÀûÓÃ×¨ÒµÍ³¼ÆÈí¼þ²úÉúµÄ·Ö×éËæ»úºÅÒÀ´Î½øÈë¸÷ÊÔÑé×é£¬²»µÃÌøºÅ»ò×ÔÖ÷Ñ¡ÔñÒ©Îï¡£È·¶¨Èë×é»¼ÕßµÄ×é±ðºó£¬ÓÉ¹Ì¶¨µÄ2ÃûÂé×íÒ½Éú°´·½°¸¸øÒ©¡£°´ 1:1:1:1µÄ±ÈÀý£¬¼Æ»®Íê³ÉÓÐÐ§²¡Àý240Àý£¬¸÷×é·Ö±ðÎª60Àý¡£
  ±¸×¢£ºÂé×íÒ½ÉúÄÜÔÙÔÚÉÏ¼¶Ò½Ê¦Ö¸µ¼ÏÂ¿ÉÕ¹¿ªASA·Ö¼¶Ⅰ~ Ⅲ¼¶²¡ÈËµÄÂé×íÈçÒ»¶þÈý¼¶ÊÖÊõÂé×í¡¢ÊÖÊõÊÒÍâÂé×í¡¢¸ßÁä²¡ÈËÂé×í£¬ÊìÁ·ÕÆÎÕÂé×íÇ°¶Ô²¡ÈËµÄÆÀ¹À£¬Âé×íÇ°×¼±¸£¬¼à²â¼¼Êõ£¬ÆøµÀ¹ÜÀí¡¢»úÐµÍ¨Æø¡¢ÒºÌå¸´ËÕ¡¢ÐÄ·ÎÄÔ¸´ËÕ£¬¾ßÓÐ¿ìËÙÕï¶ÏºÍ´¦ÀíÄÜÁ¦¡£
Ò©ÎïÅäÖÆ£º
Êæ·ÒÌ«Äá
1Ö§£¨50ug£©  50mlNS  1ug/ml       10ml¿ÕÕë
ÄÉ²¼·È
   
1Ö§£¨20mg£© 20mlNS  1mg/ml     È¡10ml        10ml¿ÕÕë   1mg/ml
                                È¡5ml
1Ö§£¨20mg£© 10mlNS  2mg/ml     È¡5ml     10ml¿ÕÕë    1.5mg/ml
                                È¡5ml ¼Ó5mlNS 10ml¿ÕÕë    1mg/ml
                                    È¡10ml       10ml¿ÕÕë    2mg/ml


¸øÒ©·½°¸£º
ËùÓÐ»¼ÕßÊõÇ°½ûÊ³8h£¬½ûË®2h£¬ÎÞÆäËûÊõÇ°ÓÃÒ©¡£»¼ÕßÈëÊÒºóÓÃ22GÁôÖÃÕëÔÚ»¼ÕßÓÒ±Û½¨Á¢¾²ÂöÍ¨µÀ£¬±Çµ¼¹ÜÎüÑõ£¨ÑõÁ÷Á¿Îª4~5L/min£©£¬³£¹æ¼à²âECG¡¢SpO2¡¢NBP¡¢RR¡¢BIS£¬×ó±Û×öÑªÑ¹²âÁ¿´¦£¬±¸ÓÃÂé×í»ú¡¢¼òÒ×ºôÎüÆ÷¡¢ÇÀ¾ÈÒ©µÈ¡£·Ö±ðÔÚÂé×íÓÕµ¼Ê±»ºÂý¾²ÂöÍÆ×¢Êæ·ÒÌ«Äá£¨0.1ug/kg£©»òÄÉ²¼·È£¨0.1mg/kg¡¢0.15mg/kg¡¢0.2mg/kg£©£¬Ëæºó¾²Âö»ºÍÆ£¨1ml/5Ãë£©±û²´·Ó40mg£¨£¼60kg£©£¬»ò50mg(£¾60kg),´ýBIS½µÖÁ80ÒÔÏÂ£¬»¼Õß½ÞÃ«·´ÉäÏûÊ§ºó¿ªÊ¼½á³¦¾µ¼ì²é¡£ÈôBIS½Ó½ü80»ò»¼Õß³öÏÖÌå¶¯¿É×·¼Ó±û²´·Ó20mg-30mg£¬ÊõÖÐÎ¬³ÖBISÔÚ60¡«80Ö±ÖÁ¼ì²é½áÊø¡£

·Ö×é	¸øÒ©·½°¸	

S×é	-	Êæ·ÒÌ«Äá0.1 ug/kgÏ¡ÊÍ¾²ÍÆ+±û²´·Ó
	

N1×é	-	ÄÉ²¼·È0.1mg/kgÏ¡ÊÍ¾²ÍÆ+±û²´·Ó
	

N2×é	-	ÄÉ²¼·È0.15mg/kgÏ¡ÊÍ¾²ÍÆ+±û²´·Ó
	

N3×é	-	ÄÉ²¼·È0.2mg/kgÏ¡ÊÍ¾²ÍÆ+±û²´·Ó
	

2.8. 3 Î§ÊõÆÚ¹ÜÀí
2.8.3.1 »¼Õß×¼±¸¼°¼à²â 
ËùÓÐ»¼ÕßÊõÇ°½ûÊ³8h£¬½ûË®2h£¬ÎÞÆäËûÊõÇ°ÓÃÒ©¡£»¼ÕßÈëÊÒºóÓÃ22GÁôÖÃÕëÔÚ»¼ÕßÓÒ±Û½¨Á¢¾²ÂöÍ¨µÀ£¬±Çµ¼¹ÜÎüÑõ£¨ÑõÁ÷Á¿Îª5L/min£©£¬³£¹æ¼à²âECG¡¢SpO2¡¢NBP¡¢RR¡¢BIS£¬×ó±Û×öÑªÑ¹²âÁ¿´¦£¬±¸ÓÃÂé×í»ú¡¢¼òÒ×ºôÎüÆ÷¡¢ÇÀ¾ÈÒ©µÈ¡£
2.8.3.2 Âé×í·½·¨
°´ÕÕ 2.8.2 ·Ö×é±íÖÐµÄ·½·¨ÊµÊ©Âé×í¡£
2.8.3.3 ºôÎüÑ­»·µÄ¼à²âÓëÎ¬³Ö
1 ²Ù×÷ÖÐÐÄÂÊ < 50´Î/min£¬×ÃÇé¾²Âö×¢ÉäÊÊÁ¿°¢ÍÐÆ·¡£
2 ²Ù×÷ÖÐÊÕËõÑ¹(SBP) < ÊõÇ°30£¥»ò80mmHg£¬¿É¾²Âö×¢ÉäÂé»Æ¼î3-5mg¡£
3 ÈôÂö²«ÑªÑõ±¥ºÍ¶È£¨SpO2) < 95%¡¢Ê±¼ä>30 sÐÐÊÖÍÐ»¼ÕßÏÂò¢£¬ÒÔ¸ÄÉÆÍ¨Æø£¬¹Û²ì²¢¼ÇÂ¼Ñõ±¥ºÍ¶ÈµÄ±ä»¯£»Èç< 90%£¬¾­¼òÒ×ºôÎüÄÒÃæÕÖ¸¨ÖúÍ¨Æø£¬¹Û²ì²¢¼ÇÂ¼Ñõ±¥ºÍ¶ÈµÄ±ä»¯¹ý³Ì¡£
4 ¼ÇÂ¼Ò©ÎïµÈµÄÊ¹ÓÃ¼ÁÁ¿¼°ÆäËûÌØÊâÓÃÒ©¡£
2.8.3.4 Ïû»¯ÏµÍ³ 
Êõºó»¼ÕßÈç³öÏÖ¶ñÐÄ¡¢Å»ÍÂ£¬¿ÉÒÔ¸øÓè¸ñÀ­Ë¾ÇíµÈÒ©Îï¶ÔÖ¢´¦Àí¡£
2.9 ¹Û²ìÖ¸±ê 
¼ÇÂ¼ÒÔÏÂÏà¹Ø¹Û²ìÖ¸±ê£¬Â¼Èë CRF ±í
2.9.1 Ò» °ãÖ¸ ±ê 
ÄêÁä¡¢Éí¸ß¡¢ÐÔ±ð¡¢ÌåÖØ¡¢ÑªÑ¹¡¢ÐÄÂÊ¡¢ÐÄµçÍ¼¡¢¼ÈÍùÊ·¡¢Âé×íÊ·¡¢ Õï¶Ï
2.9.2 Ö÷ ÒªÖ¸ ±ê 
»ùÓÚÈçÏÂ3Ïî±äÁ¿µÄ3·ÖÁ¿±í£¬ÆÀ¹À³¦¾µ²åÈë¡¢¹ýÆ¢Çú¡¢¹ý¸ÎÇúÌõ¼þ
1. µÖ¿¹²åÈë£ºÎÞ/ÇáÎ¢/Ã÷ÏÔ£»
2. Ãæ²¿±íÇé£ºÎÞ/ÇáÎ¢±ä»¯/Í´¿à£»
3. Ö«Ìå/Í·²¿ÔË¶¯£ºÎÞ/ÇáÎ¢/Ã÷ÏÔ
      2.9.3 ´ÎÒªÖ¸ ±ê 
  2.9.3.1Ê±¼ä
¿ªÊ¼¸øÊæ·ÒÌ«Äá»òÄÉ²¼·È¿ªÊ¼ÓÃÃë±í¼ÇÂ¼Ê±¼ä£¬¼ÇÂ¼ÓÕµ¼Ê±¼ä¡¢ËÕÐÑÊ±¼ä£¨ÕöÑÛÊ±¼ä¡¢°´Ö¸ÁîÍê³É¶¯×÷Ê±¼ä¡¢ÀëÊÒÊ±¼ä£©
2.9.3.2±û²´·ÓÓÃÁ¿£¨ÓÕµ¼¼ÁÁ¿¡¢Î¬³Ö¼ÁÁ¿£©
2.9.3.4³¦Èä¶¯ÆµÂÊ Âý£¨²»Ó°Ïì¼ì²é£©/¿ì£¨Ó°Ïì¼ì²é£©
2.9.3.5ÉúÃüÌåÕ÷
ÑªÑ¹£¨BP£©¡¢ÐÄÂÊ£¨HR£©¡¢ÂöÑõ±¥ºÍ¶È£¨SPO2%£©£¨ÓÕµ¼Ç°¡¢ÓÕµ¼ºó1min¡¢2min¡¢3min¡¢6min¡¢9min¡¢12min¡¢15min¡¢18min¡¢21min¡¢24min¡¢27min¡¢30min£©
     2.9.3.6 ÌÛÍ´ÆÀ·Ö£¨VASÆÀ·Ö£©
     VASÆÀ·Ö·½·¨
      ÓÃÒ»Ìõ³¤10cm µÄÓÎ¶¯±ê³ß£¬±êÓÐ10¸ö¿Ì¶È£¬Á½¶Ë·Ö±ðÎª0·ÖºÍ10·Ö£¬ÈÃ²¡ÈË ÔÚÖ±³ßÉÏ±ê³öÄÜ´ú±í×Ô¼ºÌÛÍ´³Ì¶ÈµÄÏàÓ¦Î»ÖÃ£¬Ëæ·ÃÕß¸ù¾Ý²¡ÈË±ê³öµÄÎ»ÖÃÎªÆäÆÀ·Ö¡£VASÆÀ·Ö·¶Î§Îª0 ¡« 10 ·Ö: 0 ·Ö±íÊ¾ÎÞÍ´£¬1¡« 3 ·Ö±íÊ¾ÇáÎ¢ÌÛÍ´£¬4¡« 6 ·Ö±íÊ¾ÖÐ¶ÈÌÛÍ´£¬7¡« 9 ·Ö±íÊ¾ÖØ¶ÈÌÛÍ´£¬ 10 ·Ö±íÊ¾×î¾çÁÒÌÛÍ´¡£
      ÁôÖÃÕë´©´ÌÊ±ÆÀ¹ÀVASÆÀ·Ö×÷Îª»ù´¡VAS£»ÇåÐÑºóÔÙÆÀ¹À
2.9.3.7²»Á¼ÊÂ¼þ
         ¼ì²éÖÐ£ºµÍÑªÑ¹¡¢µÍÑõ±¥ºÍ¶È¡¢ÆøµÀ¹£×è¡¢ºôÎüÒÖÖÆ¡¢ºôÎüÔÝÍ£¡¢ÐÄ¶¯¹ý»º¡¢ÐÄ¶¯¹ýËÙ¡¢Ìå¶¯
         ¼ì²éºó¼°ÀëÊÒºó24HÄÚ£ºÀä/ÈÈ¡¢¶ñÐÄ¡¢Å»ÍÂ¡¢ÊõÖÐÖªÏþ¡¢Ø¬ÃÎ/ÐÀÔÃ¡¢ÊÈË¯/Ñ£ÔÎ¡¢Æ¤·ôðþÑ÷¡¢Ë¤µ¹
 2.9.3.8ÂúÒâ¶È
      »¼Õß£»ÄÚ¾µ²Ù×÷Õß£»Âé×íÒ½Éú
2.9.4 Êõ ÖÐ ¹Û²ì 
2.9.4.1 ¼ì²éÏà¹ØÐÅÏ¢ 
¼ì²éÆðÖ¹Ê±¼ä£¨¼ì²éÊ±¼ä·ÅÈë³¦¾µÈ¡³ö³¦¾µ£©¡¢ ÒÔ¿ªÊ¼¸ø±û²´·ÓµÄÊ±¼äÎªÂé×í¿ªÊ¼Ê±¼ä£¬ÕâÊ±¿ªÊ¼ÓÃÃë±í¼ÆÊ±£¬¼ÇÂ¼ËæºóµÄ½ÞÃ«·´ÉäÏûÊ§Ê±¼ä¡¢½ø¾µÊ±¼ä¡¢×·¼ÓÒ©ÎïÊ±¼ä¡¢ËÕÐÑÊ±¼ä¡¢×ÔÓÉ»î¶¯Ê±¼äºÍÀëÔºÊ±¼ä¡£±¸×¢£¨²Ù×÷Ïà¹ØÊÂ¼þ£º1 È¡»î¼ì£»2 ¸ü»»ÊõÕß£»3 µ¥´¿³¦¾µ¼ì²é£»4 ³¦Ï¢ÈâÇÐ³ýÊõ£»5²Ù×÷ÄÑ¶È´ó£¬ÐèÒª¸¨Öú°´Ñ¹ÏÂ¸¹£©¡£
2.9.4.2 Âé×íÏà¹ØÐÅÏ¢
£¨1£©	ÓÃÒ©——ÓÕµ¼£»×·¼Ó£»ÓÃÒ©¼ÁÁ¿¼°Ê±¼ä£»
£¨2£©	¼à²â——Ñ­»·£¨SBP/DBP¡¢MAP¡¢HR£©£»ºôÎü£¨ºôÎüÆµÂÊ¡¢SpO2¡¢ÉÏºôÎüµÀ¹£×è£©£»
£¨3£© Ð§ÄÜ——³¦¾µ½ø¾µË³Àû¡£
2.9.4.3 ²»Á¼ÊÂ¼þ·¢Éú·Ö¼¶¼°´¦Àí
£¨1£©µÍÑªÑ¹£¨0/1/2£©£º0¼¶£ºSBP>ÊõÇ° 70£¥»ò 80/50mmHg£»1¼¶£º SBP<ÊõÇ° 70£¥»ò 80/50mmHg Îª 1£¬2¼¶£ºSBP<ÊõÇ° 60£¥»ò70/40mmHg
£¨2£©ÐÄÂÊÓëÐÄÂÉ±ä»¯£¨0/1£©£ºÐÄÂÊÐ¡ÓÚ 50 ´Î/·Ö£»´óÓÚ 120 ´Î/·Ö;»ò³öÏÖÐÄÂÉÊ§³£Îª 1£¬·´Ö®Îª 0¡£
£¨3£©µÍÑõÑªÖ¢·Ö¼¶£¨1/2/3/4£©£º0 ¼¶£º96%~100%£»1 ¼¶£º91%~95%£»2 ¼¶£º86%~90%£»3 ¼¶£ºµÍÓÚ85%
£¨4£©ÉÏºôÎüµÀ¹£×è£¨0/1/2£©£º0 ¼¶£ºÎÞÉÏºôÎüµÀ¹£×è£»1 ¼¶£ºÇáÎ¢÷ýÉùµ«Î¬³ÖÕý³£SPO2£»2 ¼¶£ººÜÇ¿µÄ÷ýÉù£¬±ØÐëÒÀÀµ¿ÚÑÊÍ¨ÆøµÀ»òÍÐÏÂò¢²ÅÄÜ»º½â£¬»ò²»ÄÜÎ¬³ÖÕý³£SPO2
£¨5£©ºôÎüÒÖÖÆ/ºôÎüÔÝÍ££¨0/1/2£©£º0¼¶£ººôÎüÆµÂÊ>12´Î/·Ö£»1¼¶£º6´Î/·Ö<ºôÎüÆµÂÊ<12´Î/·Ö£»2¼¶£ººôÎüÆµÂÊ<6´Î/·Ö
£¨6£© Ìå¶¯£¨0/1/2£©£º£¨°üÀ¨¸ÉÅ»ÍÌÑÊ£¬ËÄÖ«»î¶¯£©£º0 ·Ö£ºÎÞÌå¶¯£»1 ·Ö£ºÒ»°ãÌå¶¯£º ½ÅÖº¶¯¡¢ÊÖ¶¯£¬²»Ó°Ïì¼ì²éµÄÌå¶¯£»2 ·Ö£ºÑÏÖØÌå¶¯£ºÍÈ¶¯»òÍÎ¶¯£¬Ó°Ïì¼ì²éµÄÌå¶¯
2.9.5 Êõ ºó¹Û ²ìÖ¸ ±ê 
2.9.5.1 ÀëÔº±ê×¼ÆÀ·Ö— Aldrete ¸ÄÁ¼ÆÀ·Ö£¨Modified Aldrete Score£©

Aldrete ¸ÄÁ¼ÆÀ·Ö	
»î¶¯Á¦     □ 2=ËÄÖ«»î¶¯×ÔÈç»òÌý´ÓÖ¸Áî
□ 1=Á½¸öÖ«ÌåÄÜ¶¯
□ 0=ËÄÖ«¶¼²»ÄÜ¶¯	
ºôÎü	     □ 2=ÄÜ×öÉîºôÎüºÍÓÐÐ§¿ÈËÔ
□ 1=ºôÎüÀ§ÄÑ¡¢±íÇ³»òÊÜÏÞ	
Ñ­»·	     □ 2=Âé×íÇ° Bp±20mmHg
□ 1=Âé×íÇ° Bp±20-50mmHg
□ 0=Âé×íÇ° Bp±50mmHg	
ÒâÊ¶  	     □ 2=ÍêÈ«ÇåÐÑ
□ 1=ÄÜ»½ÐÑ
□ 0=ÎÞ·´Ó¦	
Ñõ±¥ºÍ¶È   □ 2=ºôÎü¿ÕÆøÊ± SpO2>92%
□ 1=Îü O2 Ê± SpO2>92%
□ 0=Îü O2 Ê± SpO2<92%	
×Ü·Ö£º	

2.9.5.2 ²»Á¼ÊÂ¼þ
£¨1£© Àä/ÈÈ£º·¢Àä»ò·¢ÈÈ¡£
£¨2£© ¶ñÐÄÅ»ÍÂ£º¶ñÐÄÅ»ÍÂÃ÷ÏÔÕßÓèÒÔ¸ñÀ­Ë¾Çí¡£
£¨3£© ÊõÖÐÖªÏþ£ºµ÷²éÊ±»ú°üÀ¨ÀëÔºÇ°µÄÊ±¼äµã¡£²ÉÓÃ¹ú¼ÊÉÏÍ¨ÓÃµÄ 5 ¾ä»°
——ÔÚÈëË¯Ç°ÄãËù¼ÇµÃµÄ×îºóÒ»¼þÊÂÊÇÊ²Ã´£¿ÔÚÐÑÀ´Ê±ÄãËù¼ÇµÃµÄµÚÒ»¼þ ÊÂÊÇÊ²Ã´£¿ÔÚÕâÁ½Õß¼äÄã»¹¼ÇµÃÊ²Ã´£¿ÔÚÊÖÊõÖÐÄã×ö¹ýÃÎÂð£¿ÓÐ¹ØÕâ´ÎÊÖ Êõ£¬Äã¸Ð¾õ×î²îµÄÊÇÊ²Ã´£¿
¸ù¾ÝÉÏÊö»Ø´ð½á¹ûÅÐ¶ÏÊÇ·ñ·¢ÉúÊõÖÐÖªÏþ¡£
£¨4£© ÆäËû£¨Ø¬ÃÎ/ÐÀÔÃ¡¢ÊÈË¯/Ñ£ÔÎ¡¢Æ¤·ôðþÑ÷¡¢Ë¤µ¹£©¡£
2.9.5.3 ÂúÒâ¶È
°üÀ¨Âé×íÂúÒâ¶È£¬ÊõÕß²Ù×÷ÂúÒâ¶ÈµÄÆÀ¹À£»»¼ÕßÂúÒâ¶È¼°Ö÷ÒªÎÊÌâ¡£°´²î/Ò»°ã/ÂúÒâ/ºÜÂúÒâÅÐ¶¨¡£

3.ÁÙ´²ÊÔÑé¼ÇÂ¼
1) È«²¿²¡Àý¾ù°´ÒÔÉÏ·½°¸¹Û²ì£¬ÈÏÕæÌîÐ´²¡Àý¼ÇÂ¼±í¸ñ¡£
2) ²¡Àý±¨¸æ±í×÷ÎªÔ­Ê¼¼ÇÂ¼£¬²»µÃÈÎÒâÍ¿¸Ä£¬Èç¹ûÌîÐ´³öÏÖ´íÎó£¬ÇëÓÃÁ½µÀ Ïß»®È¥´íÎó£¬ÖØÐÂÌîÈëÕýÈ·Êý¾Ý»òÕß²ÉÓÃ¸½¼ÓËµÃ÷µÄ·½Ê½£¬Ç©Êð¸üÕýÈËÐÕ ÃûµÄÆ´ÒôËõÐ´ºÍ¸üÕýÈÕÆÚ¡£²»ÒªÓÃÏðÆ¤²Á¡¢ÐÞÕýÒºµÈ·½Ê½ÑÚ¸ÇÌîÈëµÄÔ­Ê¼ Êý¾Ý¡£
3) ÁÙ´²ÊÔÑéÖÐÊÔÑéÊÒÊý¾Ý¾ùÓ¦¼ÇÂ¼¡£
4) ¶ÔÒì³£Êý¾ÝÐë¼ÓÒÔºËÊµ£¬ÓÉ²Î¼ÓÁÙ´²ÊÔÑéµÄÒ½Ê¦ÅÐ¶ÏÊÇ·ñ¾ßÓÐÁÙ´²ÒâÒå£¬ ÈçÓÐÁÙ´²ÒâÒå£¬Ó¦¼ÇÂ¼²»Á¼ÊÂ¼þ£¬²¢×ö±ØÒªËµÃ÷¡£
5) ÌîÐ´²¡Àý±¨¸æ±íÒ»ÂÉÊ¹ÓÃÀ¶ºÚÄ«Ë®»òºÚÉ«Ç©×Ö±Ê¡£
4.ÊÔÑéÊý¾ÝµÄÍ³¼Æ·ÖÎö

4.1.1 Ñù±¾Á¿¼ÆËã

±¾ÊÔÑé¸ù¾Ý×îµÍÂö²«ÑªÑõ±¥ºÍ¶È½øÐÐÑù±¾Á¿¹À¼Æ£¬ÊÔÑé²ÉÓÃÑôÐÔ¶ÔÕÕ£¬Í³¼Æ·ÖÎö²ÉÓÃ·½²î·ÖÎö»ò·Ç²ÎÊý¼ìÑé¡£¹À¼Æ¹«Ê½Îªn=2[(Z1-α£«Z1-β)(S/δ)]2£¬ÆäÖÐαÎª0.025£¬βÎª0.2¡£¸ù¾ÝÎÄÏ××ÊÁÏ£¬Á½×é¼ä×îµÍÑªÑõ±¥ºÍ¶È²îÖµÔ¼Îªδ=4%£¬S=6.93£¬¼ÆËãËùµÃÃ¿×éÀýÊýÎª48Àý¡£¿¼ÂÇµ½ÍÑÂäÂÊµÈÒòËØ£¬±¾´ÎÁÙ´²ÊÔÑéÉè¼ÆÃ¿×é60Àý£¬¹²¼Æ240Àý
4.1.2 È« ·ÖÎö ¼¯£¨ FAS£¬ full analysis set£© 	
°´ ITT£¨Intention-to-treat£©Ô­Ôò£¬ËùÓÐ¾­Ëæ»ú»¯·Ö×é£¬ÖÁÉÙÊ¹ÓÃ¹ýÒ»´ÎÑÐ¾¿Æ·¡¢ÇÒ¾ßÓÐÓÃÒ©ºóÆÀ¼ÛÊý¾ÝµÄ²¡Àý£¬¹¹³É±¾ÑÐ¾¿µÄ FAS¡£FAS ÖÐÁÆÐ§Ïà¹Ø²¿·ÖµÄÈ± Ê§Êý¾Ý½«²ÉÓÃÖ®Ç°×îºóÒ»´Î¹Û²âÊý¾Ý½á×ªµÄ·½·¨½øÐÐ²¹³ä¡£FAS ÊÇÁÆÐ§ÆÀ¼ÛµÄÖ÷Òª ÈËÈº¡£
4.1.3 ·û ºÏ·½ °¸¼¯ £¨ PPS£¬ per protocol set £© 
PPS ÊÜÊÔÕß·ûºÏÊÔÑé·½°¸¹æ¶¨µÄÈëÑ¡±ê×¼,Íê³ÉÈ«²¿¹Û²ìÆÚ¼Æ»®£»ÊÔÑéÆÚ¼äÎ´Ê¹ÓÃ¿ÉÄÜÓ°ÏìÁÆÐ§ÆÀ¼ÛµÄÆäËûÒ©Îï»òÖÎÁÆ´ëÊ©¡£PPS ÊÇÁÆÐ§ÆÀ¼ÛµÄ´ÎÒªÈËÈº¡£
4.1.4°² È«ÐÔ ·ÖÎö Êý¾Ý¼¯ £¨ SS£¬ safety set £© 

¾­ÊÜÊÔÕßËæ»ú»¯·Ö×éºó£¬ÖÁÉÙÊ¹ÓÃÒ»´ÎÑÐ¾¿Ò©Îï¡¢¾ßÓÐÓÃÒ©ºó°²È«ÐÔÆÀ¼ÛÊý¾ÝµÄÈ«²¿²¡Àý£¬¹¹³É±¾ÑÐ¾¿µÄ°²È«ÐÔ·ÖÎöÈËÈº¡£

4.2 ÁÆÐ§ºÍ°²È«ÐÔÖ¸±ê
4.2.1 Ö÷ ÒªÆÀ ¼ÛÖ¸ ±ê 
a) °²È«ÐÔÖ¸±ê£ºÑªÑ¹£»
b) °²È«ÐÔÖ¸±ê£ºÑªÑõ±¥ºÍ¶È£»
C) ÁÆÐ§ÐÔÖ¸±ê£º»¼Õß·ûºÏÀëÔº±ê×¼µÄÊ±¼ä£»
4.2.2 ´Î ÒªÆÀ ¼ÛÖ¸ ±ê 
a) ²»Á¼ÊÂ¼þµÄ·¢ÉúÂÊ	b) ¾­¼ÃÑ§Ö¸±ê
4.2.3 Æä ËûÆÀ ¼ÛÖ¸ ±ê 
¸÷ÖÖÂé×í¿ÉÄÜ³öÏÖµÄ²»Á¼ÊÂ¼þÓë²»Á¼·´Ó¦£¬ÒÔ¼°ÓÃÒ©ºóÁÙ´²Ö÷ÒªÖ¢×´µÄÒì³£±ä»¯¡¢ÓÐÁÙ´²ÒâÒåµÄÊÔÑéÊÒ¼ì²éµÄÒì³£¸Ä±äµÈ¡£

4.3 Í³¼Æ·ÖÎö·½·¨
4.3.1 Ò» °ãÔ­ Ôò 
ÊÔÑé×éÓë¶ÔÕÕ×éÖÎÁÆ½áÊøºó£¬Ö÷ÒªÁÆÐ§Ö¸±êµÄ×é¼ä±È½Ï²ÉÓÃÓÅÐ§ÐÔ¼ìÑé£¬P≤ 0.05 ÈÏÎªÓÅÐ§³ÉÁ¢¡£ÆäËûËùÓÐÍ³¼Æ¼ìÑé¾ùÎªË«²à¼ìÑé£¬P≤0.05 ¿ÉÈÏÎª²îÒìÓÐÍ³¼Æ Ñ§ÒâÒå¡£
Í³¼ÆÑ§ÒâÒå£ºÖ÷ÒªÆÀ¼ÛÖ¸±êÑªÑ¹ÏÂ½µµÄ·¢ÉúÂÊ£¬Á½×éÖ®¼äÓÐ 30%µÄ²îÒì¡£
¶¨Á¿Êý¾Ý£º²ÉÓÃËãÊõÆ½¾ùÊý¡¢ÖÐÎ»Êý¡¢±ê×¼²îºÍ Min ¼° Max ½øÐÐÍ³¼ÆÃèÊö¡£ 
·ÖÀàÊý¾Ý£º²ÉÓÃÆµÊý¡¢¹¹³É±È»ò°Ù·Ö±È½øÐÐÍ³¼ÆÃèÊö¡£
Êý¾Ý¿âÓëÍ³¼Æ·ÖÎö: ÓÃ SPSS 21.0 Í³¼ÆÈí¼þ½øÐÐÍ³¼Æ·ÖÎö¡£
4.3.2 ²¡ ÀýÌØ Õ÷ 	
Èë×é¼°Íê³ÉÇé¿ö£º×Ü½áÈë×é¼°Íê³É²¡ÀýÊý£¬ÁÐ³öÍÑÂä²¡ÀýµÄÇåµ¥¡£ 
Ò»°ãÐÅÏ¢µÄ»ùÏßÌØÕ÷: »ùÏß¶¨ÒåÎª²¡ÀýÉ¸Ñ¡ÆÚËù»ñµÃµÄÊý¾Ý¡£¶Ô»¼ÕßµÄÈË¿ÚÑ§ÌØÕ÷¡¢ Ö¢×´ÌåÕ÷¡¢ºÏ²¢Ö¢¡¢¹ýÃôÊ·¡¢²¡Ê·µÈ½øÐÐÃèÊö¡£Ò»°ã»ùÏß×ÊÁÏÆÀ¼ÛÕë¶Ô FAS ½øÐÐ¡£
4.3.3 ÁÆ Ð§ÆÀ ¼Û 	
    ÓëÁÆÐ§ÆÀ¼ÛÏà¹ØµÄÖ¸±êÔÚÉ¸Ñ¡ÆÚËù»ñµÃµÄÊý¾Ý¶¨ÒåÎª»ùÏßÊý¾Ý¡£°´ FAS¡¢PPS ½øÐÐ»ùÏßÆÀ¼Û¡£·ÖÀà×ÊÁÏµÄ×é¼ä±È½Ï²ÉÓÃÖÈºÍ¼ìÑé£¨µÈ¼¶×ÊÁÏ£©¡£¼ÆÁ¿×ÊÁÏµÄ×é¼ä±È½Ï²ÉÓÃµ¥ÒòËØ·½²î·ÖÎö»òÖÈºÍ¼ìÑé¡£ P≤0.05 ÈÏÎªÓÅÐ§³ÉÁ¢¡£
4.3.4 °² È«ÐÔ ÆÀ¼Û 
① ¶Ô¸÷ÖÎÁÆ×éµÄ²»Á¼ÊÂ¼þ·Ö±ðÍ³¼Æ·¢ÉúÀýÊý£¬Àà±ðºÍÑÏÖØ³Ì¶È¡£
② MH-χ2 ¼ìÑé»ò Fisher’s ¾«È·¸ÅÂÊ·¨±È½ÏËÄ×é²»Á¼ÊÂ¼þ·¢ÉúÂÊ¡£
③ µ¥ÒòËØ·½²î·ÖÎö±È½ÏËÄ×éÁ¬Ðø±äÁ¿µÄÊÔÑéÊÒ¼ì²éÖ¸±ê¡£
④ Í³¼ÆÃèÊöÈý×éÖÎÁÆºóÊÔÑéÊÒÖ¸±êÕý¡¢Òì³£±ä»¯µÄ±ÈÀý¡£
5. ÊÔÑé¹ÜÀí
 5.1 ÉêÃ÷
´ËÏîÁÙ´²ÊÔÑé½«»áÑÏ¸ñ×ñÊØ·½°¸ºÍ·¨¹æÀ´½øÐÐ
  5.2Â×Àí²¿·Ö
   ×ñÑ­ºÕ¶ûÐÁ»ùÐûÑÔ£¨2000Äê°æ£©°´ÕÕÖÐ¹úÓÐ¹ØÒ½Ñ§ÑÐ¾¿¹æ·¶¡¢·¨¹æ½øÐÐ¡£ÔÚÑÐ¾¿¿ªÊ¼Ö®Ç°£¬ÓÉÒ½Ñ§ÑÐ¾¿¸ºÔðµ¥Î»µÄÂ×ÀíÎ¯Ô±»áÅú×¼¸ÃÊÔÑé·½°¸ºó·½¿ÉÊµÊ©ÁÙ´²ÊÔÑé¡£Ã¿Ò»Î»²¡ÈËÈëÑ¡±¾ÑÐ¾¿Ç°£¬ÑÐ¾¿Ò½Ê¦ÓÐÔðÈÎÒÔÊéÃæÎÄ×ÖÐÎÊ½£¬ÏòÆä»òÆäÖ¸¶¨´ú±íÍêÕû¡¢È«ÃæµØ½éÉÜ±¾ÑÐ¾¿µÄÄ¿µÄ¡¢³ÌÐòºÍ¿ÉÄÜµÄ·çÏÕ¡£Ó¦ÈÃ»¼ÕßÖªµÀËûÃÇÓÐÈ¨ËæÊ±ÍË³ö±¾ÑÐ¾¿¡£ÈëÑ¡Ç°Ðë¸øÃ¿Î»»¼ÕßÒ»·ÝÊéÃæ»¼ÕßÖªÇéÍ¬ÒâÊé£¨ÒÔ¸½Â¼ÐÎÊ½°üÀ¨ÓÚ·½°¸ÖÐ£©£¬ÑÐ¾¿Ò½Ê¦ÓÐÔðÈÎÔÚÃ¿Î»»¼Õß½øÈËÑÐ¾¿Ö®Ç°»ñµÃÖªÇéÍ¬Òâ£¬ÖªÇéÍ¬ÒâÖÐÓ¦×÷ÎªÁÙ´²ÊÔÑéÎÄµµ±£Áô±¸²é¡£ 
 5.3Ô­Ê¼Êý¾ÝºËÊµ
   ¶ÔÖ±½Ó¼ÇÂ¼ÓÚ²¡Àý±¨¸æ±íÉÏµÄÊý¾Ý£¨¼´Ô¤ÏÈÎÞÊéÃæµÄ»òµç×Ó¼ÇÂ¼µÄÊý¾Ý£©ÒÔ¼°¿¼ÂÇÎªÔ­Ê¼Êý¾ÝµÄ¼ø¶¨£¬¸ù¾Ý·½°¸ÊÂÏÈÒªÔÚ¼à²é¼Æ»®ÖÐ×÷³ö¹æ¶¨£¬Ã÷È·ËµÃ÷£¬·ñÔòÊÓÎªÈ±·¦Ô­Ê¼Êý¾Ý¡£
  ÑÐ¾¿Õß±ØÐëÍ×ÉÆ´¦ÀíËùÓÐÁÙ´²ÑÐ¾¿¹ý³ÌÖÐ»ñµÃµÄÊý¾Ý£¬ÒÔ±£Ö¤²ÎÓëÁÙ´²ÑÐ¾¿µÄÊÜÊÔÕßµÄÈ¨ÀûºÍÒþË½¡£ÑÐ¾¿Õß±ØÐëÍ¬Òâ¼à²éÔ±/»ü²éÔ±/ÊÓ²ìÔ±¶ÔËùÐèÒªµÄÁÙ´²ÑÐ¾¿×ÊÁÏ½øÐÐ²éÔÄºÍÉóºË£¬ÒÔ±ãÑéÖ¤Ô­Ê¼×ÊÁÏµÄ×¼È·ÐÔºÍÁË½âÑÐ¾¿µÄ½øÕ¹Çé¿ö¡£Èç¹û²»ÄÜ¶ÔÔ­Ê¼¼ÇÂ¼½øÐÐÑéÖ¤£¬ÔòÑÐ¾¿ÕßÓ¦Í¬ÒâÐ­Öú¼à²éÔ±/»ü²éÔ±/ÊÓ²ìÔ±¶ÔÊý¾ÝµÄÖÊÁ¿½øÐÐ½øÒ»²½µÄÈ·ÈÏ¡£
5.4 ÖÊÁ¿¿ØÖÆºÍ±£Ö¤
±¾ÑÐ¾¿ÎªÇ°Õ°¡¢Ëæ»ú¡¢Ë«Ã¤ÊÔÑé£¬ÊÔÑéÑÐ¾¿Éè¼Æ¡¢·½°¸ÊµÊ©ÓÉ×¨ÈË¸ºÔð£¬±¾ÈË²»²ÎÓëÊý¾Ý·ÖÎö£¬±ÜÃâÈËÎªÒòËØ¸ÉÈÅÊÔÑé½á¹û¡£
5.5 ÖªÇéÍ¬ÒâÊé/Êý¾Ý±£»¤Ð­Òé
     ÑÐ¾¿ÕßÓÐÔðÈÎ¶ÔÃ¿¸öÊÜÊÔÕß½âÊÍ±¾´ÎÁÙ´²ÊÔÑéµÄÄ¿µÄ¡¢·½·¨¡¢Òæ´¦ºÍÇ±ÔÚ·çÏÕ£¬²¢»ñµÃÁÙ´²ÊÔÑéµÄÊÜÊÔÕßÇ©ÊðµÄÖªÇéÍ¬ÒâÊé¡£ÔÚÈÎºÎÓëÁÙ´²ÊÔÑéÏà¹ØµÄ²Ù×÷³ÌÐò¿ªÊ¼Ö®Ç°£¬±ØÐë»ñµÃÊÜÊÔÕßµÄÖªÇéÍ¬ÒâÊé¡£¶ÔÓÚÄÇÐ©ÒòÈÎºÎÔ­Òò¶ø²»ÄÜ×Ô¼ºÇ©ÊðÖªÇéÍ¬ÒâÊéµÄÊÜÊÔÕßÀ´Ëµ£¬±ØÐëÓÉ·¨¶¨´úÀíÈËÇ©ÊðÖªÇéÍ¬ÒâÊé¡£Í¨¹ýÇ©ÊðÖªÇéÍ¬ÒâÊé£¬ÊÜÊÔÕß»¹±ØÐëÍ¬ÒâÔÊÐíÁÙ´²ÑÐ¾¿µÄ¼à²éÔ±/»ü²éÔ±/½¡¿µµ÷²é×éÖ¯¶ÔÒÑ»ñµÃµÄÓÐ¹ØÁÙ´²ÑÐ¾¿µÄÔ­Ê¼Êý¾Ý×ÊÁÏ½øÐÐºË²é£¬ÒÔ±ãÈ·¶¨ÁÙ´²Êý¾Ý½á¹ûµÄ¿É¿¿ÐÔ¡£
5.6²»Á¼ÊÂ¼þ¹Û²ì¡¢¼ÇÂ¼ºÍ´¦ÖÃ
   ±¾ÊÔÑé½«ÓÐ×¨ÈË¼ÇÂ¼CRF±í£¬ÏêÏ¸¼ÇÂ¼Õû¸öÊÔÑé¹ý³ÌÖÐµÄ²¢·¢Ö¢¡£Õû¸öÊÔÑé¹ý³ÌÓÐÑÏ¸ñµÄ²»Á¼ÊÂ¼þ¹Û²ì¼ÇÂ¼±í£¬Ò»µ©·¢ÉúÈÎºÎ²»Á¼ÊÂ¼þ£¬Á¢¼´ÉÏ±¨ÊÔÑé¸ºÔðÈË¼°¿ÆÊÒ¡£Èç¹ûÔÚÁÙ´²ÊÔÑéÖÐ³öÏÖ²»¿ÉÔ¤ÖªµÄ²»Á¼·´Ó¦£¬ÎÒ½«µÃµ½Ò½ÉúºÍÉê°ìµ¥Î»Í×ÉÆ»ý¼«µÄÃâ·ÑÖÎÁÆ£¬Èç¹û·¢ÉúÓëÑÐ¾¿Ò©ÎïÓÐ¹ØµÄÑÏÖØ²»Á¼ÊÂ¼þ£¬³ýµÃµ½Í×ÉÆ»ý¼«µÄÃâ·ÑÖÎÁÆÍâ£¬Éê°ìÕß½«»á¸ºÔðÓÉ´ËÒýÆðµÄÏà¹ØÖÎÁÆ·ÑÓÃ¼°Åâ³¥¡£
5.7Ïà¹Ø·ÑÓÃ
    ±¾ÑÐ¾¿ÖÐÏà¹Ø¼ì²âÎªÎÞÍ´³¦¾µ¼ì²éÖÐ²âÑªÑ¹¡¢ÐÄµçÍ¼¼°Âö²«Ñõ±¥ºÍ¶È£¬¾ùÎª³£¹æ¼à²â,ÄÔµçË«ÆµÖ¸Êý¼ì²â·Ñ¼°ÄÉ²¼·È×¢ÉäÒº·ÑÓÃÓÃÓÉÉê°ìÕß³Ðµ£¡£±¾ÑÐ¾¿Ëæ·ÃÎªÎÞÍ´³¦¾µ¼ì²éºó»Ö¸´ÊÒ¹Û²ì£¬ÀëÔººó24Ð¡Ê±»ò48Ð¡Ê±Ò»´Î»òÁ½´Îµç»°Ëæ·Ã£¬ÎÞ¶îÍâËæ·Ã·ÑÓÃ¡£
6. ÁÙ´²ÊÔÑé¸ºÔðÈËÇ©×Ö±í
ÐÕÃû	µ¥Î»	Ö°³Æ	ÁªÏµµç»°	Ç©×Ö	
		 			
		 			
Éê°ì·½ÁÙ´²ÊÔÑé¸ºÔðÈË
ÐÕÃû	µ¥Î»	ÁªÏµµç»°	Ç©×Ö	
				
7.¸½Â¼£º
7.1 ²Î¿¼ÎÄÏ×£º
[1] ÖÐ¹úÏû»¯ÄÚ¾µÕïÁÆÕò¾²"Âé×íµÄ×¨¼Ò¹²Ê¶, ÁÙ´²Âé×íÑ§ÔÓÖ¾, 09 (2014) 920-927.
[2] O.H. Salman, A controlled, double blind, study of adding Nalbuphine to Propofol for laryngeal mask insertion conditions and hemodynamics in adults, Egyptian Journal of Anaesthesia, 31 (2015) 277-281.
[3] E.V.G. R. FOURNIER, M.MACKSAY and Z. GAMULIN, Onset and offset of intrathecal morphine versus nalbuphine for postoperative pain relief after total hip replacement, Acta anaesthesiologica Scandinavica, 44 (2000) 940-945.
[4] ÓáÑô, ÃÅÕïÊÖÊõÂé×íÒ©µÄÑ¡Ôñ, ¹úÍâÒ½Ñ§.Âé×íÑ§Óë¸´ËÕ·Ö²á, (1992) 319.
[5] ²ÌÁ¢æº, ÕÅ¿¡, ÅíÎÄÐË, ÖìÈÙ»ª, ÍõÐãÃ·, Ñô½£, ÕÅ†¢ÖÇ, ÄÉ²¼·È×¢ÉäÒºÔÚ½¡¿µÊÜÊÔÕßÖÐµÄÒ©´ú¶¯Á¦Ñ§ÑÐ¾¿, ÖÐ¹úÒ©Ñ§ÔÓÖ¾, (2011) 1597-1600.
[6] Àè»·, µØ×ôÐÁ¡¢Âð·È¡¢ÄÉ²¼·È¶ÔÓÚÃÅÕï»¼ÕßÊõºóÕòÍ´Ð§¹ûµÄ±È½Ï, ÖÐ¹úÒ½ÔºÒ©Ñ§ÔÓÖ¾, (2013) 978-981.
[7] M.Z. Anna Kubica-Cielińska, The use of nalbuphine in paediatric anaesthesia, Anaesthesiology Intensive Therapy, 47 (2015) 252-256.
7.2ÖªÇéÍ¬ÒâÊé
