# Supplementary material for: Comparison of nalbuphine and sufentanil for colonoscopy: A randomized controlled trial
Source: PLoS One. 2017 Dec 12;12(12):e0188901. doi: 10.1371/journal.pone.0188901 (PMC5726642; doi:10.1371/journal.pone.0188901)
Supplement: S3 File — (DOC) [file pone.0188901.s003.doc]

          Protocol
Equivalent doses study of nalbuphine and sufentanil for colonoscopy under bispectral index monitoring


Date£º       2015.11.20
                          
                          Trial center:  Sichuan University West China Hospital
                          Department£º Department of Anesthesiology
                         Study leader£º     Xiao Wang    PhD


The abstract of protocol
Title	Equivalent doses study of nalbuphine and sufentanil for colonoscopy under bispectral index monitoring	
Edition/Date	2.0  / 2015.11.20	
Objectives
	The efficacy and safety of four groups undergoing colonoscopy will be evaluated :
S 0.1ug/kg sufentanil+ propofol£»
N1 0.1mg/kg nalbuphine+ propofol£»
N2 0.15mg/kg nalbuphine+ propofol£»
N3 0.2mg/kgnalbuphine+ propofol.	
Trial design	We’ll carry out a prospective, randomized, and doubled-blinded clinical trial 	
Sample size	240 patients	
Duration 	About three months	
Participants	1.	Eligibility criteria for participants
(i) aged 18-65 years;
(ii) Body Mass Index(BMI) 18.5-30 kg/m2; 
(iii) American Society of Anesthesiologists(ASA) classification of I or II; 
(iv) the duration time of colonoscopy£¼30min.£»
Exclusion criteria included: 
(i) history of abnormal anesthesia recovery; 
(ii) Electrocardiograph(ECG): heart rate (HR)£¼60 times/min; 
(iii) Systolic blood pressure(SBP)£¾180mmHg or SBP£¼90mmHg; (iv) acute airway inflammation in the past 2 weeks; 
(v) neuromuscular disease; 
(vi) possible or diagnosed difficult airway; 
(vii) suspected abuse of narcotic analgesics or sedatives; 
(viii) history of allergy to propofol or opioids; 
(ix) unable to communicate.	
Drugs in trial	Nalbuphine hydrochloride 20mg/2ml
Sufentanil   50ug/1ml
Propofol 200mg/20ml	

Allocation	S 0.1ug/kg sufentanil+ propofol£»n=60
N1 0.1mg/kg nalbuphine+propofol£»n=60
N2 0.15mg/kg nalbuphine+ propofol£»n=60
N3 0.2mg/kgnalbuphine+ propofol£»n=60	
Methods	After obtaining informed consent, patients were randomly assigned to one of four groups.	
Efficacy and safety indicators	Main evaluation index
Validity index: Evaluation of colonoscopyinsert, splenic flexure,hepatic flexure conditions(smooth)
1.Resistance to insert: no/mild/obvious; 2.The facial expression: no/mild/obvious; 3. The body/head movement: no/light/obvious.
The secondary evaluation index
1.	Time: began with a stopwatch to record time,record the induction time, wake up of time, normal life and work.
2.	The total propofol dose¡¢frequency of bowel movements
3.	Baseline vital signs£ºBlood pressure¡¢Heart rate¡¢
oxygen saturation(SPO2%)£¨every 1min for the first 3 min after induction and every 3 min thereafter£©
visual analogue score(VAS)£¨ before the procedure and in the Post Anesthesia Care Unit(PACU)£©
4.	Side effects£º
     Intraoperation:hypotension, low oxygen saturation, airway obstruction, respiratory depression, apnea, bradycardia, tachycardia, body movement.    
Postoeration: cold/hot, nausea, vomit, nightmare, drowsiness, puritus, abdominal pain.
5.	Satisfaction£ºpatients; physicians; anesthetist	
Statistical Analysis	1£®Statistical Analysis
1.1  FAS£¬ full analysis set:According to the principle of ITT(Intention-to-treat),all the randomized groups,using at least one study medicine,with cases of evaluated data, which constitute FAS. Curative effect in the relevant part of the missing data will adopt the method of the last observation data before carry forward to supplement.FAS is curative effect evaluation of the main groups.
1.2  PPS£¬ per protocol set£ºThe participants meet the inclusion criteria of the requirements of protocal,and complete the whole observation period plan;During the trial there will be no other treatments or drugs affecting the curative effect.PPS is the secondary groups of curative effect evaluation.
1.3 SS£¬ safety set£ºAfter randomized grouping,participants will use at least one study drugs, and possess all the cases of drug safety evaluation data, which constitute the safety analysis of the present study population.
 2.Statistical analysis method
Use SPSS21.0 statistical software for statistical analysis.
Baseline assessment:The index associated with efficacy in screening data defined as baseline data.  In accordance with the FAS,PPS base line evaluation. Classification of data comparison between groups using rank and inspection data(level).Measurement data of comparison between groups using single factor analysis of variance and rank and inspection.when P≤0.05,superior effect was established.	

Flow chart of the study design.


           
Contents    
1.Backgroud and objectives	1
2.Trial design	2
2.1 Trial drugs	2
2.2 The title	2
2.3 The place	2
2.4 Time	2
2.5 Methods	2
2.6 The inclusion and exclusion criteria
2.6.1 Inclusion criteria	2
2.6.2 Exclusion criteria	3
2.6.3 exit criteria	3
2.7 Personnel requirements	4
2.8 Methods	4
2.8.1 The drugs	4
2.8.2 The experimental group and the dosage regimen	4
2.8.3  Perioperative management	5
2.9 Observation index	6
2.9.1 general index	6
2.9.2 main index	6
2.9.3 secondary index	6
2.9.4 Intraoperative observation index	7
2.9.5 Postoperative observation index	8
3.Clinical trail records	9
4.Statistical analysis of  trial dada	9
4.1 	9
	FAS£¬full analysis set	9
	 PPS£¬per protocol set 	9
4.1.3 SS£¬safety set 	9
4.2 Efficacy and safety index	9
4.2.1 main evaluation index	9
4.2.2 secondary evaluation index	9
4.2.3 other evaluation index	9
4.3 Statistical Analysis	10
4.3.1 general principle	10
4.3.2 Characteristics of cases	10
4.3.3 Therapeutic evaluation	10
4.3.4 Safety evaluation	10
5.Trial management	10
6.Signature table	11
7.Appendix	11
	
1.	Backgroud and objectives  
Colonoscopy is the most important and reliable method for the diagnosis and treatment of lower digestive tract diseases. But colonoscopy long operation time, strong stimulation, especially the bowel gas injection and was pulling the hand, nausea, pain, and even intestinal loop or intestinal spasm, elevated blood pressure, heart rate, and even induce angina, myocardial infarction, stroke or cardiac arrest and other serious complications; on the other hand, bring tension anxiety and fear to patients, a few patients can not tolerate and with the completion of endoscopic procedures, so that doctors cannot clearly endoscopic diagnosis and treatment of related diseases. At present, a lot of units in our country have already carried out sedation and anesthesia in endoscopic diagnosis and treatment. Endoscopy of the sedation / anesthesia is through the application of sedative and narcotic analgesics and related technology, to eliminate or reduce endoscopy or during the treatment of pain, abdominal distension, nausea and vomiting and other subjective pain and discomfort, especially can eliminate the fear of patients to check again, to improve the patients of digestive endoscopy acceptance, and create better conditions for the diagnosis and treatment of endoscopic doctor.[1]
             At present the common clinical moderate or deep sedation, intravenous injection of fentanyl or sufentanil (30~50ug) (3~5ug) and (or) a small dose of midazolam (1~2mg), and then given 1~2mg/kg of propofol or etomidate 0.2~0.3mg/kg. The patient's spontaneous breathing was slow but steady, the eyelash reflex disappeared, the whole body muscles relaxed, and the jaw was unresponsive. If the diagnosis and treatment for a long time or the operation of a strong stimulus, according to patients with signs such as deep breathing, heart rate, and body movement, every vein of propofol or etomidate 0.2~0.5mg/kg 0.1mg/kg, until the end of inspection. [1] propofol has an inhibitory effect on the circulation and upper airway reflex and leads to the patients with no intention to exercise. So, do not recommend medication as independent, clinical application combined with opioids and propofol with high success rate, but respiratory depression, apnea, hypotension, dizziness, abdominal distension and pain after the examination is still the main side effects.[2]
                     Nalbuphine hydrochloride [(-) -17 (cyclobutylcarbinyl) -4,5 alpha epoxy morphinans -3,6 alpha, 14- three ethanol hydrochloride is kappa agonist] / mu receptor antagonist analgesics, pain control and morphine, for the treatment and prevention of moderate to severe pain. No cardiovascular side effects of nalbuphine, respiratory inhibition is also slightly, and the ceiling effect. Nalbuphine usually 2 ~ 3 minutes of onset, 30 minutes to reach the peak, can maintain 3 ~ 6 hours analgesia, ceiling effect dose of 0.3 ~ 0 5mg /kg.
              Foreign studies show that intrathecal nalbuphine combined with morphine reduces the side effects of opioids and does not affect postoperative analgesia. [3] can also be associated with the high proportion of bupivacaine intrathecal nalbuphine and propofol for surgical abdomen, urinary system and lower extremity induction for laryngeal mask airway insertion of [2] on domestic nalbuphine little research, Yu Yang said in 1992 that nalbuphine for outpatient anesthesia. Study on pharmacokinetics of [4]2011 Cai Lijing of nalbuphine injection in healthy subjects. [5]2013 Li ring compared dezocine and morphine and nalbuphine for patients with postoperative analgesic effect. [6]
             Nalbuphine is not in the excitement of narcotic drugs and psychotropic drugs in the list, can be used as non narcotic drugs excited storage, and based on the basis of routine prescription application. [7] this is also expected to solve the problem of the use of propofol for sedation in our country due to the restriction of fentanyl family. In addition, because of its unique role and mechanism of moderate sedation analgesia, preoperative application of nalbuphine 0.1-0.2mg/kg can relieve the pain, bring calm and stability.
        Therefore, this study is mainly to observe and compare the analgesic effect of sufentanil for painless colonoscopy and nalbuphine and safety, to explore the optimal dosage of nalbuphine for the painless colonoscopy. In the process of using propofol and sufentanil after clinical application shows that the long-term safety of narcotic drugs is classic, nalbuphine injection for domestic marketed drugs, the clinical application proved to be safe and reliable.
2.Trial Design
2.1 Trial drugs
Sufentanil£ºProduction from yichang renfu pharmaceutical co,.LTD£¬50ug/1ml.
Nalbuhine£ºProduction from yichang renfu pharmaceutical co,.LTD£¬20mg/2ml¡£
Propofol£ºProduction by astrazeneca pharmaceutical co.,LTD,200mg/20ml
2.2 Trial Title 
Equivalent doses study of nalbuphine and sufentanil for colonoscopy under bispectral index monitoring
2.3 Trial Institution
Sichuan University West China Hospital
2.4 Trial period 
Start time  2015.12  	End time  2016.12  
2.5 Trial method
A prospective, randomized, and doubled-blinded clinical trial 
2.6 Inclusion , exclusion, exit criteria
2.6.1 Inclusion criteria
(i) aged 18-65 years;
(ii) Body Mass Index(BMI) 18.5-30 kg/m2; 
(iii) American Society of Anesthesiologists(ASA) classification of I or II; 
(iv) the duration time of colonoscopy£¼30min.£»
2.6.2 Exclusion criteria
(i) history of abnormal anesthesia recovery; 
(ii) Electrocardiograph(ECG): heart rate (HR)£¼60 times/min; 
(iii) Systolic blood pressure(SBP)£¾180mmHg or SBP£¼90mmHg; (iv) acute airway inflammation in the past 2 weeks; 
(v) neuromuscular disease; 
(vi) possible or diagnosed difficult airway; 
(vii) suspected abuse of narcotic analgesics or sedatives; 
(viii) history of allergy to propofol or opioids; 
(ix) unable to communicate.
2.6.3 Exit criteria
2.6.3.1 Excluding criteria
With the already into the group of cases but one of the following, should be excluded
1	No inspection record£»
2	Case report on the record is not complete.
	3 lost to follow up£»
4	 the duration time of colonoscopy£¾30min¡£ 
Excluding cases should give a reason,corresponding medical and research records shall be retained for future reference.Don’t make a statistical analysis,but at least a treatment ,and a safety record, as the case may participate in safety analysis. 
2.6.3.2 Shedding criteria
Not completed for the following reasons regard as shedding cases £º
1 Participants can’t complete the trial because of  adverse events.
    2 Poor adherence£»
    3 During the study period participants use some inhibited drugs so that we can’t make a efficacy and safety evaluation.
2.7 Personnel requirements
	Surgery doctor fixed two; pharmacy fixed one; Anesthesiologists fixed one; observers fixed one.
2.8 Trial method
2.8.1 Trial drugs 

Common name	manufacturers	Brand name	Specifications	
Sufentail	Yichang renfu pharmaceutical co.,LTD 		50ug/1ml	
Nalbuphine	Yichang renfu pharmaceutical co.,LTD 	 ruijing	20mg/2ml	
Propofol	Astrazeneca pharmaceutical co.,LTD	Diprivan	200mg/20ml	
2.8.2 Allocation
patients were randomly assigned to one of four groups by  statistical software. The shall not use number ir choose their drugs. 
Drug preparation£º
Sufentanil one£¨50ug£©  50mlNS  1ug/ml       10ml empty needle
Nalbuphine
   one£¨20mg£© 20mlNS  1mg/ml     10ml        10ml empty needle       1mg/ml
                                                                  5ml
one£¨20mg£© 10mlNS  2mg/ml     5ml     10m lempty needle      1.5mg/ml
                                
                                                             5ml   5mlNS   10mlempty needle       1mg/ml
                                                                       10ml            10mlempty needle         2mg/ml


Dosage regimen£º
All the patients fast 8h,water 2h before trial,and no other preoperative medication. With 22G needle to build venous channel in their right arm and All patients were given supplemental oxygen intranasally (5 L/min) and continuous monitoring for HR (three-lead electrocardiogram), oxygen saturation (pulse oximetry), blood pressure (automated blood pressure cuff, serial measurements every 3 minutes) , SpO2, BIS ( BIS vista monitoring system)  Respiratory rate(RR) and end-tidal CO2(ETCO2).easy backup anesthesia machine, ventilator,rescue,etc.
Patients received either sufentanil or nalbuphine.  Propofol was initially administered at a rate of 1ml (10mg)/5 seconds to maximum dose of 4 ml(40mg), if body weight£¼60kg, or 5ml(50 mg), if body weight£¾60kg. Additional doses (20¡«30mg) of propofol were administered if the patient began to move, or if the BIS value started rising to 80.
Allocation	Dosage regimen	
S	-	 0.1ug/kg sufentanil+ propofol	
N1	-	 0.1mg/kg nalbuphine+ propofol	
N2	-	 0.15mg/kg nalbuphine+ propofol	
N3	-	 0.2mg/kg nalbuphine+ propofol	

2.8. 3 Trial management
2.8.3.1 Prepare and mornitor
All the patients fast 8h,water 2h before trial,and no other preoperative medication. With 22G needle to build venous channel in their right arm and All patients were given supplemental oxygen intranasally (5 L/min) and continuous monitoring for HR , blood pressure, BIS,RR and ETCO2.easy backup anesthesia machine, ventilator,rescue,etc.
2.8.3.2 Anesthesia method
According to the above method, start the procedure.
2.8.3.3 monitor and maintain
1.Atropine (0.3-0.5mg) iv was administered in cases where the heart rate decreased to £¼50 bpm.  
2. Ephedrine(3-5mg) was administered to treat arterial hypotension, which was defined as SBP of 80mmHg or a reduction in SBP£¾30%, compared with baseline values
3 Airway maneuvers(i.e. jaw thrust and chin lift) woud be manipulated, if the RD cases happened
4 Record the drug dosage and other special drugs
2.8.3.4 Digestive system
If there are nausea or vomiting during postoperation,we can give granisetron,ect.
2.9 Observational index 
Record the following relevant observation indexes,and entry CRF table.
2.9.1 general index:
Age, height, weight,sex,blood pressure,heart rate and blood test and ECG (if have), past medical history, history of anesthesia and the diagnosis.
2.9.2 Main evaluation index
     Evaluation of colonoscopy insert,  splenic flexure, hepatic flexure conditions(smooth)
1. Resistance to insert: no/mild/obvious; 2.The facial expression: no/mild/obvious; 
3. The body/head movement: no/light/obvious.
      2.9.3 The secondary evaluation index
2.9.3.1Time
Time: began with a stopwatch to record time,record the induction time, wake up of time, normal life and work.
2.9.3.2The total propofol dose£¨induction¡¢maintaince£©
2.9.3.4frequency of bowel movements
2.9.3.5Baseline vital signs£º
Blood pressure¡¢Heart rate¡¢
oxygen saturation(SPO2%)£¨every 1min for the first 3 min after induction and every 3 min thereafter£©
     2.9.3.6 VAS
     VAS score method
      Use a scale of 10cm long,with10 scale both ends are respectively 0 points and 10 points, to let the patients in the ruler can represent their pain degree of the corresponding position, along with the visitors according to mask the location of the patient for the score. VAS score range of 0-10;0mean painless,1-3 points mean slight pain£¬4¡« 6 points mean moderate pain, and 7-9 points mean severe pain 
      Record VAS  before the procedure and then in the Post Anesthesia Care Unit.
2.9.3.7 Side effects
 Intraoperation:hypotension, low oxygen saturation, airway obstruction, respiratory depression, apnea, bradycardia, tachycardia, body movement.    
Postoeration: cold/hot, nausea, vomit, nightmare, drowsiness, puritus, abdominal pain.

 2.9.3.8Satisfaction£ºpatients; physicians; anesthetist
      
2.9.5Postoperative observation index
2.9.5.1 Modified Aldrete Score

Modified Aldrete Score	
Activity    □ 2=Able to move 4 extremities voluntarily or on command
□ 1=Able to move 2 extremities voluntarily or on command
□ 0=Able to move 0 extremities voluntarily or on command	
Respiration	     □ 2=Able to deep breathe and cough freely
□ 1=Dyspnea or limited breathing
□ 0=Apneic	
Circulation     □ 2=BP +/- 20% of Preanesthetic level
□ 1=BP +/- 20-50% of Preanesthetic level
□ 0=BP +/- 50% of Preanesthetic level	
Consciousness 	     □ 2=Fully Awake
□ 1=Arousable on calling
□ 0=Not responding	
Color  □ 2=Pink
□ 1=Pale, dusky blotchy, jaundiced, other
□ 0=Cyanotic	
Total points£º	

2.9.5.2 Adverse events
(1) Cold/hot
(2) Nausea, vomit£ºwe can give granisetron,ect.
          (3) Nightmare, drowsiness, puritus, abdominal pain.

2.9.5.3 Satisfaction
patients;physicians;anesthetist. According to the poor/ general/ very satisfied with their decision.

3. Clinical trail records
1) All cases according to the above observation, fill in case record form.
2) Case report form as the original records, and may not  arbitrarily alter, if fill in error,please use two line rule out mistakes, to fill in the correct data or use additional instructions, sign the correct name and correct data. Don’t use the eraser, correction fluid, etc way to fill in the cover of the raw data.
3) Clinical trial data shall be recorded.
4) verify abnormal data and physicians have to determine whether clinical significance, if any, shall be recorded adverse events and make the necessary instructions.
5) Fill in the form cases use the blue-black ink or black pen.
4.Statistical Analysis
4.1.1 Sample calculation

This test according to the minimun pulse oxygen saturation sample size estimation, positive control was used with statistical analysis using analysis of variance or nonparametic test. Estimated formula for n=2[(Z1-alpha+Z1-beta)(S/g)]2,the alpha 0.025,beta of 0.2.according to the literature,the lowest oxygen saturation difference between the two groups about the delta=4%,S=6.93, compute the number of cases in each group of 48 cases. Considing the loss rate and other factors,the clinical trial design 60 cases in each group, atatal of 240.
4.1.2  FAS£¬ full analysis set 	
According to the principle of ITT(Intention-to-treat),all the randomized groups,using at least one study medicine,with cases of evaluated data, which constitute FAS. Curative effect in the relevant part of the missing data will adopt the method of the last observation data before carry forward to supplement.FAS is curative effect evaluation of the main groups.
4.1.3 PPS£¬ per protocol set  
The participants meet the inclusion criteria of the requirements of protocal,and complete the whole observation period plan;During the trial there will be no other treatments or drugs affecting the curative effect.PPS is the secondary groups of curative effect evaluation.
4.1.4 SS£¬ safety set
     After randomized grouping,participants will use at least one study drugs, and possess all the cases of drug safety evaluation data, which constitute the safety analysis of the present study population.

4.2 Efficacy and safety index
4.2.1 Main evaluation index
    a) safety index£ºblood pressure£»
b) safety index£ºoxygen saturation£»
c) Index of curative effect: Time when departure from the hospital£»
4.2.2 Secondary evaluation index
a) incidence of side effect  b) Economic index
4.2.3  Other evaluation index
Various possible adverse events and adverse reactions,and clnic main symptoms after the changes and clinical significance of abnormal changes of laboratory inspection,etc.

4.3 Statistical analysis method
4.3.1 General principal
The test group and the control group after treatment, compared the main index of the curative effect between group with superiority test, P = 0.05, that is better established. All statistical tests were two-sided test, P = 0.05 can be considered statistically significant difference.
Statistical significance: the main indicators of the incidence of decreased blood pressure, there was a 30% difference between the two groups.
Quantitative data: using arithmetic mean, median, standard deviation and Min and Max for statistical description.
Classification data: frequency, composition ratio or percentage of statistical description.
Database and statistical analysis: using SPSS 21 statistical software for statistical analysis.
4.3.2 Case characteristics	
Into the group and the completion of the situation: summary of the group and the completion of the number of cases, the list of cases listed off¡£ 
Baseline characteristics of general information: baseline is defined as data obtained during the screening period. The demographic characteristics, symptoms and signs, complications, history of allergies, history and so on were described. Baseline data evaluation for FAS.
4.3.3 Efficacy evaluation	
   The data obtained from the screening period were defined as baseline data for the evaluation of the efficacy. Baseline evaluation by FAS and PPS. Comparisons between groups were performed using rank sum test. The comparison between the measured data was analyzed by one-way ANOVA or rank sum test. P is less than or equal to 0.05 that the excellent effect was established.
4.3.4 Safety evaluation
① The number, type and severity of adverse events were calculated for each treatment group.
②MH- chi square test or Fisher 's exact probability method was used to compare the incidence of adverse events between the four groups.
③ Single factor analysis of variance to compare four groups of variables in the laboratory test indicators.
④ Statistical description of the three groups after treatment of laboratory indicators positive and abnormal changes in the proportion of.
5. Trial management
 5.1 State
This trial will strictly abide by the program and regulations.
  5.2The ethical part
Following the Helsinki declaration (2000 Edition) in accordance with the relevant medical research codes and regulations of China. Prior to the start of the study, the ethics committee approved by the medical research unit will be able to carry out clinical trials. Every patient in this study, the research physicians have a responsibility to written form, to comprehensively introduces the research purpose, procedures and the possible risks. Patients should be informed that they have the right to withdraw from the study at any time. The top must give each patient a written informed consent (the appendix is included in the program), research physician has the responsibility to obtain informed consent before each patient in the study, informed consent in clinical trials should be retained for future reference document.
5.3Original data verification
To directly recorded in the case report form data (i.e. no written or electronic records in advance of data) and consider for the identification of the original data, according to the plan in advance to make provision in the monitoring plan clearly stated, otherwise regarded as lack of original data.
Researchers must properly handle all data obtained during clinical studies to ensure the rights and privacy of the participants in the clinical study. Researchers must agree with the arbitrator / Inspector / inspector of clinical research data needed for inspection and audit, in order to verify the accuracy of original data and understand the research progress. If not the original record is verified, researchers should agree to assist the arbitrator / Inspector / inspectors of the quality of the data for further confirmation.
5.4 Quality control and assurance
This study is a prospective, randomized, double blind trial, the experimental study design, implementation of the program by the person in charge, I do not participate in data analysis, to avoid the interference of human factors test results.
5.5 Informed consent / data protection protocol
It is the responsibility of the investigator to explain the purpose, methodology, benefits and potential risks of this clinical trial for each subject, and to obtain informed consent from the subjects in the clinical trial. Informed consent must be obtained prior to the start of any operational procedures related to clinical trials. For those who are unable to sign their own informed consent for any reason, it is necessary to sign an informed consent form. By signing the informed consent, participants must also agree to allow clinical research associate / Inspector / Health Survey Organization for verification has been obtained on clinical research of the original data and the reliability of data in order to determine the clinical results.
5.6Adverse event observation, recording and disposition
In this experiment, we will record the CRF table and record the complications during the whole experiment. The entire test process has a strict record of adverse events, in the event of any adverse events, the report immediately to the person in charge and the department. If adverse reactions occur unpredictably in clinical trials, I will get the doctor and unit properly active free treatment, if serious adverse events related to the study drug, in addition to properly active free treatment, treatment fees and compensation related applicant will be responsible for the resulting
5.7Relevant cost
Correlation detection in this study for painless colonoscopy to measure blood pressure, electrocardiogram and pulse oximetry, for routine monitoring of bispectral index testing fee and expenses for injection of nalbuphine by the applicant undertake. The study was followed up for 24 days or at least one hour or two hours after the observation of painless colonoscopy, and there was no additional follow-up.
6. Signature table
Name	institution	profession	Phone numbum	signature	
		 			
		 			
7.Appendix£º
7.1Reference£º
[1] ÖÐ¹úÏû»¯ÄÚ¾µÕïÁÆÕò¾²"Âé×íµÄ×¨¼Ò¹²Ê¶, ÁÙ´²Âé×íÑ§ÔÓÖ¾, 09 (2014) 920-927.
[2] O.H. Salman, A controlled, double blind, study of adding Nalbuphine to Propofol for laryngeal mask insertion conditions and hemodynamics in adults, Egyptian Journal of Anaesthesia, 31 (2015) 277-281.
[3] E.V.G. R. FOURNIER, M.MACKSAY and Z. GAMULIN, Onset and offset of intrathecal morphine versus nalbuphine for postoperative pain relief after total hip replacement, Acta anaesthesiologica Scandinavica, 44 (2000) 940-945.
[4] ÓáÑô, ÃÅÕïÊÖÊõÂé×íÒ©µÄÑ¡Ôñ, ¹úÍâÒ½Ñ§.Âé×íÑ§Óë¸´ËÕ·Ö²á, (1992) 319.
[5] ²ÌÁ¢æº, ÕÅ¿¡, ÅíÎÄÐË, ÖìÈÙ»ª, ÍõÐãÃ·, Ñô½£, ÕÅ†¢ÖÇ, ÄÉ²¼·È×¢ÉäÒºÔÚ½¡¿µÊÜÊÔÕßÖÐµÄÒ©´ú¶¯Á¦Ñ§ÑÐ¾¿, ÖÐ¹úÒ©Ñ§ÔÓÖ¾, (2011) 1597-1600.
[6] Àè»·, µØ×ôÐÁ¡¢Âð·È¡¢ÄÉ²¼·È¶ÔÓÚÃÅÕï»¼ÕßÊõºóÕòÍ´Ð§¹ûµÄ±È½Ï, ÖÐ¹úÒ½ÔºÒ©Ñ§ÔÓÖ¾, (2013) 978-981.
[7] M.Z. Anna Kubica-Cielińska, The use of nalbuphine in paediatric anaesthesia, Anaesthesiology Intensive Therapy, 47 (2015) 252-256.
7.2Informed consent
