# Supplementary material for: Comparison of nalbuphine and sufentanil for colonoscopy: A randomized controlled trial
Source: PLoS One. 2017 Dec 12;12(12):e0188901. doi: 10.1371/journal.pone.0188901 (PMC5726642; doi:10.1371/journal.pone.0188901)
Supplement: S5 File — (DOC) [file pone.0188901.s005.doc]

**Bioethics board approval documents review of West China hospital of Sichuan university**

2016 (34)

| Department | Anesthesiology | Name of study derector | Xiao Wang |
| --- | --- | --- | --- |
| Title | Equivalent doses study of nalbuphine and sufentanil for colonoscopy under bispectral index monitoring | | |
| Protocol | Edition 2.0 | Date of edition | 2015.11.20 |
| Concent form | Edition - | Date of edition | - |
| Review opinion:  The qualification of researcher meets ethical requirements.  The study protocol and consent form accord with ethical requirements.  Review result: □Agree □Agree after necessary amendment  □Retrial after necessary amendment □Disagree □Terminate or suspend    Institution(seal):  Chairman(signature):  February 12, 2016 | | | |
