# Supplementary figures and images for: Comparison of nalbuphine and sufentanil for colonoscopy: A randomized controlled trial
Source: PLoS One. 2017 Dec 12;12(12):e0188901. doi: 10.1371/journal.pone.0188901 (PMC5726642; doi:10.1371/journal.pone.0188901)

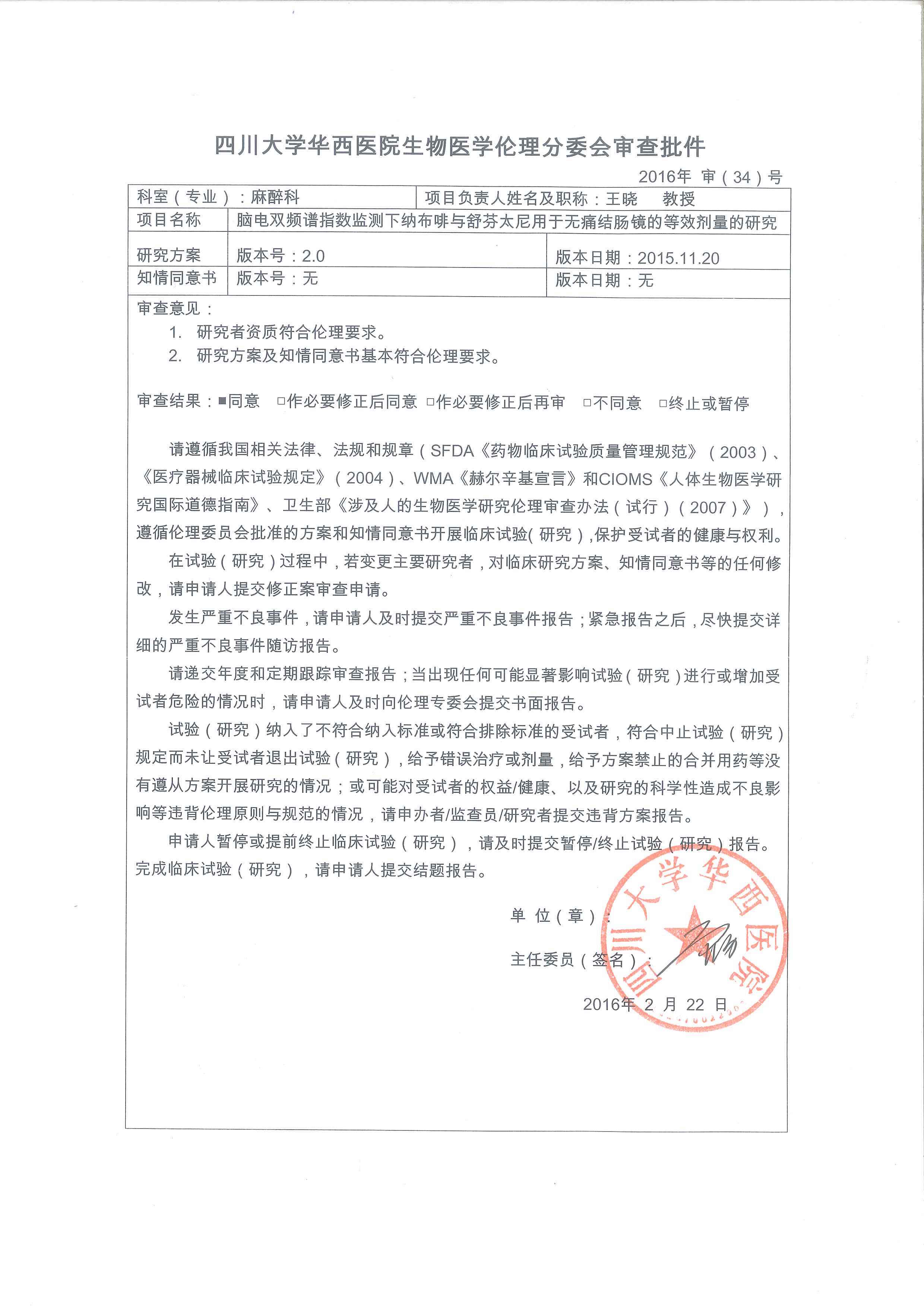

Supplement: S1 Fig — (JPG) [file pone.0188901.s006.jpg]
